# Day-to-day experiences and challenges of long-term warfarin use among patients with prosthetic heart valves at a National Cardiac Institute in Tanzania: A qualitative study

**DOI:** 10.1371/journal.pone.0355506

**Published:** 2026-08-05

**Authors:** Benedicto T. Mgala, Salome E. Buluba, Aisha A. Omar, Dickson A. Mkoka

**Affiliations:** 1 Department of Clinical Nursing, Muhimbili University of Health and Allied Sciences, Dar es Salaam, Tanzania; 2 Department of Nursing Services, Jakaya Kikwete Cardiac Institute, Dar es Salaam, Tanzania; University of Kwazulu-Natal, SOUTH AFRICA

## Abstract

**Background:**

Long-term warfarin therapy following prosthetic heart valve placement is essential for preventing valve-related thromboembolic complications. Due to its narrow therapeutic window, maintaining a therapeutic International Normalized Ratio (INR) requires strict adherence to healthcare medication, dietary recommendations, and regular monitoring. Despite receiving education at discharge, patients with prosthetic heart valves continue to demonstrate poor adherence to warfarin therapy. While previous quantitative studies have identified factors associated with non-adherence, little is known about patients’ day-to-day experiences and challenges with long-term warfarin use in low-resource settings.

**Aim of the study:**

This study explored day-to-day experiences and challenges encountered during long-term warfarin use among patients with prosthetic heart valves attending a National Cardiac Institute in Dar es Salaam, Tanzania.

**Materials and methods:**

An explorative qualitative study design using inductive content analysis was conducted among twelve (12) patients with prosthetic valves on long-term warfarin therapy, who were purposively recruited based on the principle of data saturation between April 2025 and June 2025 at a national cardiac institute. In-depth interviews were conducted using a semi-structured interview guide. Data were analyzed manually using a deductive-inductive content analysis approach.

**Results:**

Two main categories emerged from this study. The first category, adapting to life with long-term warfarin use, describes participants’ experiences of symptom relief and improved quality of life after surgery, alongside issues related to dietary restrictions, medication side effects, emotional distress, and reduced engagement in socio-economic activities. Participants perceived dietary recommendations as restrictive and reported difficulties with lifestyle modifications required during long-term warfarin therapy. The second category, navigating warfarin care within an unstructured continuum of care, reports participants’ struggles in maintaining therapeutic INR levels amid financial difficulties, inconsistent medication use, contradictory information from healthcare providers, and limited access to INR monitoring and warfarin services in peripheral regions. Some participants reported self-adjusting warfarin doses or missing follow-up appointments due to cost and accessibility barriers.

**Conclusion and recommendations:**

Although participants experienced clinical improvement post-surgery, they continued to face substantial challenges related to long-term warfarin use and access to anticoagulation care. Strengthening patient-centered education, improving continuity and consistency of anticoagulation counselling, decentralizing warfarin and INR monitoring services, and enhancing structured long-term follow-up systems may improve warfarin adherence and patient outcomes in low-resource settings.

## Introduction

Valvular heart disease (VHD) is a leading global cause of cardiovascular symptoms and functional disability, contributing to an estimated 1.4 million deaths and over 10 million disability-adjusted life years annually [1–3]. While degenerative and functional VHD dominate in high-income countries, rheumatic heart disease (RHD) caused by rheumatic fever remains the primary form in low- and middle-income countries [4]. In 2019, the global prevalence of RHD was estimated at 40.5 million, with the highest burden among marginalized populations in sub-Saharan Africa (SSA) [2,4]. Rheumatic heart disease complicating to VHD remains highly prevalent in Tanzania, with pooled estimates around 9% and echocardiographic studies reporting subclinical valvular diseases in approximately 2–3% of school-aged children, highlighting a substantial disease burden [1,5,6].

Valvular heart diseases are generally progressive, and surgical treatment is required in cases of severe insufficiency or stenosis [7,8]. Patients need to cope with further difficulties despite successful surgery. One of these difficulties in care post-surgery is the patients’ lifelong use of anticoagulant medication [7,9,10]. Anticoagulation is crucial in preventing valve-related thromboembolic complications. It is assessed using the international normalized ratio (INR) test [7]. The target therapeutic INR value varies according to the valve type, ranging between 2.5–3.5 in the mitral valve, and 2.0–3.0 in the aortic valve [9]. A high INR value is associated with anticoagulant toxicity and poses the risk of bleeding, while a low INR value increases the risk of thrombosis to the patient [11]. In SSA, suboptimal anticoagulation control among patients on warfarin has been widely reported, further increasing the risk of both thromboembolic and bleeding complications in this population [12]. Although direct oral anticoagulants (DOACs) are increasingly used in other cardiovascular conditions, their use in patients with mechanical prosthetic heart valves remains limited due to insufficient evidence on safety and effectiveness in this population. As a result, warfarin remains the standard anticoagulant therapy for these patients. Moreover, the availability and affordability of newer anticoagulants such as DOACs remain major challenges in low-resource settings [7,13].

Warfarin is used as a long-term oral anticoagulant in patients with prosthetic heart valves in Tanzania [8]. For an effective therapeutic outcome, warfarin adherence is mandatory [10,14]. Patients are usually discharged with health information given regarding consistent medication use with the recommended dose, dietary and lifestyle modification, and regular blood tests for INR monitoring [9,12,15–17]. The health information given at discharge aims to promote warfarin compliance, prevent complications, and improve patients’ quality of life [15,18,19].

Despite the health education given upon discharge regarding the appropriate use of warfarin among these patients, various studies reported low patient adherence to warfarin use, ranging from 20% to 65% [17,19–22]. Most of these studies are quantitative in nature and have mainly identified factors associated with anticoagulant adherence, including knowledge, age, gender, heart valve surgery, alcohol consumption, and cost of treatment [19,22–25]. Few studies conducted in Tanzania have reported poor clinical outcomes among patients with prosthetic valves on long-term warfarin therapy, with limited documentation of the underlying reasons contributing to the low adherence and anticoagulation outcomes among these patients [8,26]. However, quantitative studies alone cannot adequately explain how patients experience long-term warfarin use in their daily lives, how they negotiate treatment recommendations, and why challenges to adherence persist despite receiving health education.

Furthermore, little is known about the lived day-to-day experiences and challenges encountered by patients with prosthetic heart valves on long-term warfarin therapy within the Tanzanian context. Qualitative exploration of these experiences might provide deeper insight into both patients and health system-related factors that require context-specific interventions to improve anticoagulation outcomes. Therefore, this study explored the day-to-day experiences and challenges encountered by patients with prosthetic heart valves regarding long-term warfarin use.

## Materials and methods

### Study design

An exploratory descriptive qualitative study design was employed to examine the day-to-day experiences and challenges of patients with prosthetic heart valves who were on long-term warfarin therapy following hospital discharge. This design was appropriate because the study sought to obtain detailed accounts of how patients experience, interpret, and manage long-term warfarin therapy in their everyday lives. Qualitative content analysis was used to analyze participants’ information so as to allow systematic description and interpretation of participants’ narratives while preserving the context in which their experiences occur. The analysis followed a combined inductive-deductive approach, whereby broad sensitizing domains informed the semi-structured interviews, while codes, sub-categories, and categories were developed from participants’ narratives rather than from fixed predetermined coding categories. This approach was important because it ensured that clinically relevant aspects of warfarin therapy were explored while still allowing participants’ own experiences, meanings, and challenges to shape the final findings.

### Study setting

The study was conducted at the Jakaya Kikwete Cardiac Institute (JKCI) in Dar es Salaam, Tanzania. This is a national cardiac hospital, providing cardiovascular care, training, and research services. The institute has a 157-bed capacity and serves over 1,800 outpatients and 100 inpatients weekly. The institute was selected for this study because it caters to patients from all regions of Tanzania, referred from regional and designated hospitals for advanced cardiovascular care. Valvular surgical replacement and repairs are among the procedures performed at the hospital, with approximately 20–40 patients undergoing the procedure per month. After achieving post-surgical stability, patients are discharged home with individualized health education, primarily provided by the attending physician, dietitian, and occasionally by nurses. This education focuses on adherence to prescribed compliance with warfarin therapy, including the rationale and importance of anticoagulation, taking the medication at the recommended dose at the same time each day, recognizing and managing drug-drug and food-drug interactions, identifying symptoms of potential complications, and understanding strategies to prevent such complications. Patients are also required to attend monthly outpatient clinic appointments at the same hospital whenever possible. During these visits, patient prognosis is monitored, and adherence to warfarin therapy is assessed through patient interviews and INR laboratory testing. Laboratory INR results are typically released within approximately two hours, after which the INR level is evaluated. Patients whose INR falls within the therapeutic range (INR = 2.5–3.5) are discharged home. However, those with critical INR values, either suggestive of warfarin toxicity (INR > 5.0) or an increased risk of thrombosis (INR < 2.0), may be admitted for several days for stabilization and provided with additional education on appropriate warfarin use at home after hospital discharge.

### Sample size and sampling of study participants

Participants were recruited using a purposive sampling technique guided by the principle of data saturation [27]. Maximum variation purposive sampling was used to obtain diverse experiences among participants based on age, sex, duration of warfarin use, type of prosthetic heart valve, and place of residence. Data saturation in this study was assessed iteratively during data collection and analysis after each interview through continuous review of emerging codes, categories, and meanings from participants’ narratives. This means that data collection stopped when the research team judged that information had become redundant, and no new codes, concepts, or deeper insights relevant to the study objectives were obtained through further data collection. In this study, code and meaning saturation were achieved with ten [10] participants after the research team judged that additional interviews were no longer generating new codes or substantially different meanings relevant to the study aim. An additional two [2] participants were recruited to confirm the consistency and saturability of the information obtained, making a total of twelve [12] study participants. A total of seventeen [17] eligible participants were approached for participation; however, five [5] declined due to competing personal commitments.

### Inclusion and exclusion criteria

The study included patients who had undergone heart valve surgery, had a prosthetic heart valve, had been discharged home on warfarin therapy, and were at least three months post-surgery. Participants were also required to be willing to participate in the study after providing informed consent. Patients who were critically ill or unable to participate in an in-depth interview were excluded from the study.

### Data collection method and tool

A semi-structured interview guide was used to collect data on participants’ day-to-day experiences and challenges with long-term warfarin use. The guide contained broad open-ended questions and sensitizing domains related to medication use, side effects, adherence behaviours, lifestyle modifications, motivators for adherence, perceived quality of life, education received from healthcare providers, conflicting advice, access to medication, and INR monitoring services. These domains were included from the literature and clinical experience to ensure that clinically relevant areas of long-term warfarin care were explored. However, interviews remained flexible, and participants were encouraged to describe their experiences in their own words and to raise additional issues that were important to them. Probing questions were used only when clarification or deeper explanation was needed.

Post-surgery participants who came for a monthly check-up at the clinic were consulted with the help of the nurse in-charge. Those who met the inclusion criteria and consented to take part in the study were asked for interview that was scheduled during the waiting time for blood laboratory results done to check their INR level. The waiting time is usually two hours. This period was considered the most convenient and practical time for conducting interviews because most clinic services at this facility are provided during daytime hours, and many participants travel from distant regions and usually leave immediately after receiving their results. Although waiting for INR results could potentially create anxiety among some participants, interviews were initiated only when participants appeared comfortable and willing to engage in discussion voluntarily. In addition, the interview took place in a private, quiet room to maintain confidentiality and lasted between 30 minutes and 45 minutes.

The interviews were conducted by the first author (B.T.M), a postgraduate Master of Science in Cardiovascular Nursing, using Kiswahili, a language with which both participants were conversant. At the time of data collection, B.T.M was also undertaking a clinical rotation at the outpatient department (OPD) of the study facility where study participants were receiving care. This positionality provided both advantages and potential biases, as it facilitated rapport and understanding of the clinical context but also raised the possibility of influence due to his dual role as a student and rotating clinician. To minimize this influence, participants were clearly informed that the interviews were conducted strictly for academic purposes and would not affect their clinical care. The researcher maintained an impartial and non-judgmental stance during interviews and avoided involvement in participants’ clinical decision-making during the study period. All interviews were recorded using audio digital recorders. The research assistant (A.A.O) was taking notes on the interview process and specific observations during interviews. These notes, together with non-verbal data, were expanded and triangulated during data analysis. Immediately after the first interview, data analysis was initiated, providing insight into preliminary sub-categories and emerging issues that were followed up on in subsequent interviews. The whole process of data collection lasted from April to June, 2025.

### Ethical considerations

Ethical approval for this study with **ref. No.DA.282/298/01.C/2751** was obtained from the Research and Ethics Committee of MUHAS. Permission to conduct the research with **Ref. No: AB.123/307/01L/61** was granted by the hospital management. Before participation, potential participants were thoroughly informed about the study’s objectives, procedures, potential risks and benefits, confidentiality measures, and their rights to withdraw from the study at any time without any consequences to their care. Written informed consent was obtained from all participants prior to data collection. The consent form was in Kiswahili, and for participants with limited literacy, the consent was explained verbally to ensure full comprehension before written consent was obtained. Participants’ identities were kept anonymous, and confidentiality was maintained throughout the study. Audio-recorded data were securely stored on a password-protected computer accessible only to the research team. In addition, notebooks containing field notes with key observations were securely stored in a locked cabinet to prevent unauthorized access.

### Data analysis

Qualitative content analysis guided by the work of Graneheim and Lundman was done manually to analyze the verbatim transcripts of the audio recordings [28]. The analysis was conducted using a combined inductive-deductive approach, whereby broad sensitizing domains informed the semi-structured interviews, while codes, sub-categories, and categories were developed from participants’ narratives rather than from fixed predetermined coding categories. The analysis began with the transcription of audio-recorded interviews in Kiswahili (The native spoken language used during the interview). Transcription was conducted by the Principal Investigator (PI) (B.T.M) with support from trained research assistants. The PI cross-checked all transcripts against audio recordings to ensure accuracy, capture important parts of participants’ narratives, and preserve contextual elements such as participant tones, pauses, and emphasis. Coding and analysis were conducted in the original Kiswahili transcripts to preserve meaning, and selected excerpts were later translated into English for reporting purposes [29]. Following transcription, the PI (B.T.M) immersed himself in the data by reading each transcript repeatedly to gain a comprehensive understanding of participants’ day-to-day experiences, challenges, and the overall context. Initial notes and reflections were documented to guide subsequent analysis. Next, the identification of meaning units was first carried out by the PI. These meaning units were then reviewed together with the supervisors (D.A.M and S.E.B) during scheduled discussion meetings. Through discussion and comparison with the original transcripts, a consensus was reached on the relevance and boundaries of the meaning units. The agreed meaning units were subsequently condensed by the PI to shorten the text while retaining the core meaning.

The PI generated preliminary codes from the condensed units. These codes, along with supporting excerpts, were shared with the supervisors (D.A.M and S.E.B) for independent review. The research team then met to discuss the codes, resolve discrepancies, refine code definitions, and agree on a common coding framework. This collaborative process ensured clarity, consistency, and analytical rigor. After agreement on the coding structure, similar codes were grouped into sub-categories by the PI. These sub-categories were presented to the supervisors for validation, where discussion focused on their internal homogeneity and external heterogeneity. Adjustments were made jointly until consensus was reached. Finally, the PI developed broader categories by interpreting relationships among the sub-categories and identifying the latent meanings within the data. These categories were critically reviewed, discussed, and refined together with the supervisors (D.A.M and S.E.B) to ensure that they accurately reflected participants’ narratives and addressed the research questions. The final categories were approved collectively by the research team. After the categories had been developed from participants’ narratives, Andersen’s Behavioural Model was used as an interpretive lens in the Discussion. This strengthened the interpretation by situating participants’ experiences within broader individual, social, and health-system influences on long-term warfarin use and access to anticoagulation care.

Moreover, Member checking was conducted with four purposively selected participants, representing approximately one-third of the study sample, with variation in sex, duration of warfarin use, place of residence, health insurance status, and anticoagulation status. Summarized findings were shared with them in Kiswahili to confirm whether the categories and sub-categories reflected their experiences and whether any interpretation was inaccurate or incomplete. Participants confirmed that the findings were consistent with their experiences. Minor wording clarifications were made, but no new category or sub-category emerged following member checking.

### Trustworthiness of the study

The trustworthiness of this qualitative study was ensured by adhering to four criteria by Lincoln and Guba: credibility, dependability, confirmability, and transferability [30]. In this study, credibility was ensured through triangulation of data from both the transcripts and those generated from the field notes. The first author has a prolonged engagement in a study setting, as he was a postgraduate Master of Science in Cardiovascular Nursing student undertaking clinical rotation at the study site before and during the data collection, which enabled familiarity with the context and improved understanding of participants’ experiences. He was directly involved in data collection; hence, he was able to capture the reality of those being studied. Furthermore, credibility was ensured through the purposeful recruitment of participants with diverse backgrounds and experiences in using warfarin post-prosthetic surgery.

To ensure dependability, an audit trail was maintained documenting coding decisions in the code book and sub-categories and categories development among the research team. Also, by using a gatekeeper in the recruitment of participants, interacting with them at the clinic, establishing rapport, and conducting interviews in Kiswahili, the researcher gained trust with the informants, ensuring the findings accurately reflect the data. Confirmability of the study was ensured through the provision of the details of the actual study setting, sampling criteria, data collection processes, and tools. Lastly, transferability was supported through an elaborative explanation of study context, participant characteristics, data collection procedures, and data analysis, allowing readers to assess the applicability of findings to their own settings.

## Results

The study recruited 12 participants with prosthetic heart valves who were on long-term warfarin therapy as their anticoagulation treatment. 7 out of 12 participants were females. Participants’ ages ranged between 30 and 66 years. The duration of warfarin use varied significantly, ranging from 10 months to 15 years. Regarding clinical characteristics, rheumatic heart disease was the underlying etiology among 8 participants, while 4 had degenerative valve disease. All participants had mechanical prosthetic valves, with 9 having mitral valve replacement and 3 having aortic valve replacement. Concerning anticoagulation status, only 3 participants had INR values within the therapeutic range, 3 were below the therapeutic range, and 6 were above the therapeutic range. Furthermore, five participants had previously experienced thromboembolic events. Warfarin doses ranged from 5 mg among 7 participants, 7.5 mg among 4 participants, and 10 mg in 1 participant. Additionally, 5 participants had health insurance coverage, while 7 had no insurance coverage. Moreover, four participants had hypertension (HTN), while two participants had diabetes mellitus (DM) as comorbid conditions, as shown in Table 1.

**Table 1:** Characteristics of study participants.

Participant no.	Age (Years)	Sex (Male “M” /Female “F)	Education	Occupation	Duration of warfarin use	Underlying etiology	Valve position	Recent INR results	Daily warfarin dosage (mg)	Health insurance status	History of thromboembolic events	Comorbidities
01	30	F	Primary	Housewife	12 months	RHD	Mitral	3.2	5	Yes	No	None
02	42	F	Primary	Housewife	15 years and 8 months	RHD	Mitral	4.7	7.5	No	No	None
03	41	F	College	Teacher	10 months	RHD	Mitral	2.4	5	No	Yes	None
04	65	F	Primary	Peasant	3 years	Degenerative	Mitral	11.0	10	Yes	Yes	HTN & DM
05	32	M	Primary	Unemployed	3 years	RHD	Mitral	4.1	5	No	No	No
06	32	F	Secondary	Entrepreneur	1 year and 2 months	RHD	Mitral	5.3	7.5	No	No	No
07	48	F	Primary	Housewife	6 years	RHD	Mitral	2.8	7.5	No	No	HTN
08	66	M	Masters	Economist	1 year and 6 months	Degenerative	Aortic	4.2	5	Yes	No	DM
09	63	M	Secondary	Peasant	2 years	Degenerative	Aortic	1.6	7.5	Yes	Yes	HTN
10	56	M	Primary	Peasant	6 years	RHD	Mitral	2.1	5	No	No	No
11	64	M	College	Retired Engineer	4 years	Degenerative	Aortic	3.7	5	Yes	Yes	HTN
12	38	F	Secondary	Entrepreneur	1 year and 9 months	RHD	Mitral	3.4	5	No	Yes	No

The analysis generated two broad categories that describe how patients negotiated long-term warfarin therapy after prosthetic heart valve surgery. Rather than representing isolated barriers, the categories show a continuing process in which participants tried to balance the perceived life-saving benefits of warfarin with the practical, emotional, dietary, financial, and health-system demands of lifelong anticoagulation. The first category, adapting to life with long-term warfarin use, reflects how participants accepted warfarin as necessary treatment while adjusting their eating practices, responding to perceived side effects, and negotiating changes in physical, social, and economic roles. The second category, navigating warfarin care within an unstructured continuum of care, reflects how participants attempted to maintain treatment in the context of inconsistent information, financial constraints, limited access to INR monitoring, and poor availability of warfarin in peripheral areas. Codes, sub-categories, and categories are presented in Table 2.

**Table 2:** Categories and sub-categories with selected codes describing participants’ experiences of Warfarin long-term use.

Codes	Sub-categories	Categories
• Dietary restriction practices • Restricted vegetable intake • Selected food consumption	(i) Modification and adoption of a new eating style	**1. Adapting to life with long-term warfarin use**
• Unable to work before surgery • Improved health status	(ii) Relief from clinical symptoms and improved quality of life	
• Itching all over the body • Changes in menstrual flow • Severe dizziness episodes	(iii) Discomfort and undesired changes from warfarin use	
• Reduced role functioning • Limited physical activity • Inability to perform strenuous tasks	(iv) Limited engagement in socio-economic activities post-surgery	
• Sometimes I stop • Fatigue with daily warfarin use	(i) Being tiresome and inconsistent on warfarin use	**2. Navigating warfarin care within an unstructured continuum of care**
• Lack of health insurance • Out-of-pocket payments • Self-adjustment of warfarin dose	(ii) Increased financial burden to cover treatment costs	
• Excessive self-dosing practices • Non-adherence to dietary advice	(iii) Non-adherence to proper warfarin use at home	
• Conflicting dietary instructions • Conflicting instructions • Lost for two years, no call	(iv) Contradictory information and mixed directives from health care providers	
• Only one clinic in Tanzania • Warfarin is expensive • Limited warfarin availability	(v) Centralized service and limited access to anticoagulation Care	

### Category 1: Adapting to life with long-term warfarin use

This category comprises four sub-categories: modification and adoption of a new eating style; relief from clinical symptoms and improved quality of life; discomfort and undesired changes from warfarin use; and limited engagement in socio-economic activities post-surgery.

#### Modification and adoption of a new eating style.

Participants reported receiving special dietary instructions from healthcare providers after starting warfarin therapy. Many recalled being told to reduce or regulate intake of foods known to influence blood thickness, particularly those high in vitamin K, such as leafy greens, certain fruits such as oranges and avocados, and common spices such as garlic, ginger, and lemon, as narrated by one participant below:

*"… I was advised that one avocado per week is enough, the same goes for oranges…As for leafy vegetables, I was told that I can eat them weekly, but it’s also good to have a routine, if I choose to eat one type of vegetable for that week, I can stick to that type. … there are also other types of spices such as ginger, garlic, and lemon, for which I was also given instructions of not using them "* **(P03)**

Some participants expressed frustration with the limited portions of vegetables recommended during warfarin therapy, feeling that the new dietary instructions left them less satisfied compared to their usual meals, as described by P11 below:

*"Now the vegetables themselves, for example, right now I’m told… can you imagine being given ugali with just one spoonful of amaranth greens? …. That’s what I’m struggling with. So, you see, I’m using just one spoonful, but in the past, I used to be served a full bowl with milk and a bit of meat, and I would eat ugali and feel full. But now I’m not allowed."* **(P11)**

#### Relief from clinical symptoms and improved quality of life.

Participants reported a noticeable improvement in their condition following valve replacement surgery and the initiation of warfarin therapy. They shared that before the surgery, they were unable to walk, but afterward, they experienced significant relief as described by P08 below:

*“Before, I couldn’t even walk... but after the valve replacement, I felt relieved.”* **(P08)**

Some participants reported that their ability to carry out daily tasks had improved. They reported being able to resume some household responsibilities and manage activities that were previously difficult:

*“Now I can do some of the house chores... I can even climb small hills.”* **(P07)**

#### Discomfort and undesired changes from warfarin use.

Some participants reported symptoms they began to experience after starting warfarin, including itching, dizziness, fatigue, prolonged or irregular menstrual cycles, gum bleeding, and dry mouth. They described the symptoms as new and unfamiliar, as they had not occurred before beginning the medication, as narrated by this participant:

*"…there were different things that happened at the beginning, conditions I wasn’t used to. I experienced changes in my menstrual cycle; it started occurring about twice a month, and sometimes it would last more days than I was used to."* **(P03)**

Some participants expressed uncertainty about the cause of these symptoms. They shared that they were not sure whether the effects were due to warfarin or something else. One participant narrated:

*"…I find myself scratching all over my body and then it feels like I’m swelling up… I don’t know what the problem is, whether it’s the medication or something else."* **(P01)**

Participants also reported how they responded to these symptoms. Some mentioned not seeking treatment and instead used self-care measures such as lying down. One participant said:

*“I’ve never used any medication for the dizziness... I just lie down until it goes away.”* **(P07)**

#### Limited engagement in socio-economic activities post-surgery.

Several participants reported facing challenges in resuming socio-economic roles after surgery. They explained that health providers advised them to avoid strenuous tasks. While some reported improvement in physical functioning, others reflected on earlier periods of severe illness during which even basic household activities were impossible, leading to prolonged dependency on others. One participant explained that before surgery, she was unable to perform routine household chores due to severe illness, but her condition improved significantly after surgery and initiation of warfarin, enabling her to resume normal daily activities. They said:

*"I started getting sick in 2019. I couldn’t do any housework, not even sweeping. I was admitted but didn’t get better until 2024, when I had surgery and started to use warfarin. Now I am fine. I do all the chores, everything a mother does.”* **(P01)**

*"For example, I’ve returned to work, but I still can’t handle all the responsibilities I used to manage, so some tasks have been reduced for me.”* **(P03)**

Participants described feeling set apart by others in the community, noting that during public gatherings, people would refer to them as “the one with heart disease,” which contributed to a sense of isolation as reported by P02 below:

*"…just people saying that you have a heart condition… it’s like you’re somewhat isolated. Even when there’s a community event, you’ll hear things like, ‘That one has heart disease, that one is sick with a heart problem."* **(P02)**

### Category 2: Navigating warfarin care within an unstructured continuum of care

This category comprises five sub-categories: being tiresome and inconsistent on warfarin use; increased financial burden to cover treatment costs; non-adherence of proper warfarin use at home; contradictory information and mixed directives from healthcare providers; and centralized service and limited access to anticoagulation Care.

#### Being tiresome and inconsistent on warfarin use.

Some participants reported that they sometimes stop taking the medication, especially when they feel well or forget. They said it’s not easy to remember every day, as narrated by P03 below:

*“Sometimes I stop, especially when I feel fine or when I forget. It’s not easy to keep remembering every day.”* **(P03)**

Another participant said they do take warfarin, but sometimes they get tired of using it daily. They shared that the daily routine feels like a burden, and at times they feel like giving up, as narrated below by P04:

*“…I do take it, but sometimes I really get tired, due to the burden of using it every single day. To be honest, sometimes I feel like giving up.”* **(P04)**

#### Increased financial burden to cover treatment costs.

Participants reported the financial cost of warfarin treatment and clinic follow-ups as a major challenge. Some shared that the lack of transport fare, consultation fees, and out-of-pocket costs for medicine delayed clinic attendance and sometimes interrupted medication use, especially before obtaining health insurance, as narrated by one participant:

*"…there are times when you might not have money… back in the years when I didn’t have insurance, I could stay at home for months, even up to 2 months, waiting until I got transport fare to come here. Sometimes I felt like giving up… Even now, still I have to find money for transport and also to buy warfarin as my insurance doesn’t cover."* **(P12)**

Considering the financial difficulties, participants suggested that warfarin be given free of charge, similar to how antiretroviral drugs are provided to people living with HIV, as narrated by one participant below:

*"I was suggesting that the government should consider giving us these medications for free, since we have to use them for the rest of our lives. There are other diseases where the government provides free medication; for example, people with HIV receive their medicines at no cost when they come to the clinic. We also take these drugs for life. Even if they can't be given for free, at least the cost should be reduced, because we buy them every month and have to take them daily."* **(P02)**

#### Non-adherence to proper warfarin use at home.

Participants also reported challenges with managing warfarin at home, particularly inconsistent self-regulation of prescribed doses based on personal interpretation of the regimen. One participant described alternating between full and half doses on different days as a personal strategy for use, reflecting variability in adherence practices and potential misunderstanding of prescribed dosing schedules, as reported by P12:

*"The way I take it is, for example, if I take a full tablet today, the next day I take half. So it’s half one day, full the next."* **(P12)**

Other participants reported continuing to eat vegetables they were advised to limit. They said they believed this might have contributed to fluctuations in their INR levels, especially when they came for check-ups, as narrated by P09 below:

*"I kept serving myself a small bowl of cassava and legume leaves. I started to think that maybe this was causing my INR to fluctuate as it was seen dropping whenever I came to test."* **(P09)**

#### Contradictory information and mixed directives from health care providers.

Participants reported receiving inconsistent and confusing instructions from different healthcare providers regarding warfarin use. Some explained that when seen by different clinicians or at different facilities, they were given varying directives about medication timing and dosage. One participant reported being instructed to take the medication both morning and evening, which led to an excessively high INR and eventual admission, as narrated by one participant below:

*“I was told to take it in the morning and evening for a week... I got worse, then they found my INR was 11 and told to stop using twice a day.”* **(P04)**

Other participants reported that the instructions given were often not explained, leaving them with little understanding of why the medication was taken a certain way. They described following instructions as they were told for many years, but without clarity on the purpose, as narrated by one participant below:

*“…you know, there was a time I became very ill. They asked me if I had taken the medication, and I told them yes, I took it in the morning before coming. They asked, 'Why did you take it in the morning?' I told them that I’ve always taken it in the morning, that’s how I was instructed years ago.”* **(P02)**

In addition to confusion about dosing, participants reported contradictory dietary guidance. Some said they were advised to avoid certain foods entirely, while others were told they could consume them in moderation, as narrated by this participant:

*“They say don’t eat it at all, then later another tells you to eat just a little... it’s confusing.”* **(P06)**

#### Centralized service and limited access to anticoagulation Care.

Participants reported the lack of INR testing services and the limited availability of warfarin in rural or peripheral areas. They reported that the existence of only one specialized cardiac center in the country requires them to travel long distances to access care. They said:

*“Sometimes you have to travel all the way to Dar es Salaam just to check your INR.”* **(P05)**

*“I struggled fetching warfarin in the whole of Tanga Town... they told me there’s no demand, so they don’t stock it.”* **(P09)**

Some participants reported missing follow-up appointments for several months due to the distance to accessing INR services and the unavailability of warfarin. They stated that during this time, they either continued taking warfarin without INR monitoring or stopped the medication altogether, as narrated by this participant below:

*“I could go even three months without coming here...I stop taking the medications, and sometimes I used to buy the medication from pharmacies.”* **(P12)**

## Discussion

This study aimed to explore the day-to-day experiences and challenges encountered during long-term warfarin use among patients with prosthetic heart valves at a National Cardiac Institute in Tanzania. The findings revealed a journey of adapting to life with long-term warfarin use and navigating warfarin care within an unstructured continuum of care.

The findings of this study were discussed in relation to Andersen’s behavioral model of health services use, which explains healthcare utilization and treatment adherence as interactions between individual, social, and health system factors. The model was not used to develop the interview guide, code the data, or generate categories, but rather used after analysis to help explain how participants’ warfarin-related experiences were shaped by interactions between individual factors, social circumstances, and health-system conditions. In this study, long-term warfarin adherence was influenced not only by personal motivation or knowledge, but also by enabling and constraining factors such as availability of INR monitoring, cost of transport and medication, health insurance coverage, continuity of counselling, and access to anticoagulation services outside the national referral centre. This interpretation highlights that challenges in warfarin adherence should not be understood only as patient-level non-compliance but also as a reflection of broader structural barriers within the anticoagulation care system. This framework helped explain how long-term warfarin management among patients with prosthetic heart valves is shaped by both patient-level experiences and broader health system conditions within the Tanzanian context [31].

### Acceptance, discomfort, relief, and modified life experience: A need for supportive and patient-centered care post -prosthetic valvular surgery

Participants reported major lifestyle adjustments after starting long-term warfarin therapy, particularly dietary practices. Many perceived these instructions as strict dietary restrictions that disrupted their usual dietary routines, rather than as guidance on maintaining consistent vitamin K intake by regulating intake of foods such as green vegetables, avocados, and certain spices. This experience reflects the difficulty of adapting to lifelong anticoagulation therapy while trying to preserve familiar eating habits in the Tanzanian context, where leaf vegetables are a common, affordable staple. The findings suggest that inadequate or overly restrictive dietary counselling may unintentionally contribute to fear, dissatisfaction, and poor adherence, underscoring the need for patient-centered care that integrates clinical follow-up, patient education, emotional support, and lifestyle counselling after prosthetic valvular surgery. Adhering to stable dietary patterns is essential to prevent INR fluctuations and reduce bleeding or thrombotic risks for patients on long-term warfarin therapy [7,13]. Similar concerns of dissatisfaction with dietary information from HCPs have been reported in other SSA countries, such as Ethiopia and Kenya, where gaps in patient education affected anticoagulation control [16,22]. Therefore, patient counselling should focus on culturally appropriate explanations emphasizing dietary consistency rather than food avoidance, while incorporating emotional support and the use of simple dietary guides to help patients adapt to lifestyle modifications.

Study participants also reported relief from debilitating pre-surgery symptoms such as breathlessness, fatigue, and limited physical functioning following surgery and initiation of warfarin therapy. This improvement motivated continued adherence to treatment and reflected positive adjustment to life after surgery. Similar findings have been reported in studies from Iran and Turkey, where patients reported feelings of renewed health and viewed warfarin as a protector of their second chance at life, particularly in helping them prevent prosthetic valve-related complications [9,32]. These findings suggest that integrated follow-up care involving clinical monitoring, continuous patient education, and psychological support may help sustain this positive recovery experience and promote long-term warfarin adherence.

Despite these improvements, participants also experienced discomfort and uncertainty related to long-term warfarin use, including dizziness, itching, gum bleeding, and menstrual changes. Some participants were unsure whether these symptoms were related to warfarin or other conditions, suggesting gaps in pharmacological counselling and continuity of follow-up care. Considering the challenging health system in Tanzania, where access to consistent health care provider interaction is limited, patients often self-manage or normalize such symptoms, leading to delaying appropriate care with ultimate consequences of severe adverse effects, such as warfarin toxicity necessitating hospitalization for these patients [26]. This situation highlights the urgent need for follow-up care models that extend beyond routine clinic visits to include accessible consultation pathways and patient-centered monitoring. These findings are similar to the study done in Kenya, which revealed gaps in warfarin-related knowledge, where 65.4% patients had poor knowledge on warfarin [22]. Therefore, strengthening patients’ counselling, improving follow-up systems, and fostering supportive patient-provider relationships are essential to minimize discomfort, enhance safety, and promote a more adaptive and supported long-term adherence to warfarin post-operatively.

Study participants also reported difficulty resuming their previous work duties, economic activities, or social roles despite the clinical improvements. The fear of overexertion and incomplete functional recovery was a barrier to resuming previous pre-morbid roles. This struggle reflects the broader modified life experience following long-term warfarin use post-operatively, where physical recovery does not always transform into full social and functional restoration, emphasizing the need for supportive and patient-centered care. In Tanzania’s predominantly informal, labor-intensive economy, such limitations create further challenges in the financial burden to cover treatment costs and threaten livelihoods [33]. Other studies done in Tanzania, Botswana, and Nigeria have reported similar challenges, where quality of life improvements post valvular surgery were stalled by limited socio-economic reintegration support, with the majority of patients (>50%) not resuming their previous roles [12,33,34]. These findings suggest that without structured reintegration strategies, patients may continue to experience discomfort and uncertainty despite successful surgery and proper adherence to warfarin postoperatively. Therefore, integrating rehabilitation services, HCPs-led functional assessments, and gradual return to work guidance into routine follow-up could foster acceptance, reduce distress, and support a more meaningful recovery transition for these patients

### Challenge of long-term use of warfarin: Query for a supportive system and structured continuum care within the health system

In this study, poor compliance with long-term warfarin therapy was not due to individual negligence, but rather to the faults in the health system, reflecting the broader challenge of long-term warfarin use post-valvular heart surgery. Study participants reported difficulties maintaining consistent warfarin use, including missed doses, self-adjustment of medication dosage, not following dietary instructions, and interruptions in clinic attendance due to financial issues, transport costs, and limited access to INR monitoring services. Structural barriers such as centralized anticoagulation services, medication shortages in peripheral facilities, and out-of-pocket expenditure constrained access to optimal care, particularly in resource-limited settings. Similar challenges have been reported in other SSA countries such as Botswana, Kenya, and Ethiopia, where access to INR testing and affordability of anticoagulation care remain major barriers to anticoagulation therapeutic control [11,16,22]. In contrast, in developed countries such as Turkey and the UK, there are more advanced healthcare systems, and patients are reported to benefit much from community-based anticoagulation clinics and home-based INR self-testing devices, thereby reducing guesswork among these patients [19,35]. Such models highlight the role of structured care in transforming anticoagulation management from reactive self-care to supervised, patient-centered support. In Tanzania, where specialized cardiac services remain centralized in urban areas, patients from distant regions experience substantial financial and logistical barriers in accessing follow-up care. These findings highlight the need for decentralized anticoagulation services, improved availability of warfarin in peripheral facilities, and strengthened continuity of care for patients requiring lifelong anticoagulation therapy.

This study reports a dynamic interplay between patient agency and structural constraints in the long-term use of warfarin among patients with prosthetic heart valves. Participants reported active self-management behaviors, including modification of dietary intake and medication use, reflecting efforts to navigate complex treatment demands in everyday life. Similar challenges have been reported in other sub-Saharan African countries, where suboptimal anticoagulation control and variability in adherence are common among patients on vitamin K antagonists [11,16,34] Evidence from Tanzania has also shown poor INR stability among patients on warfarin, highlighting persistent difficulties in maintaining therapeutic anticoagulation [26,34]. These challenges are partly attributable to limited patient education and inconsistent follow-up, which have been identified as important determinants of warfarin adherence in previous studies [18,25]. The coexistence of patient-driven coping strategies and health system limitations suggests that adherence is shaped by both individual and contextual factors rather than patient behavior alone. This situation highlights the need for patient-centered follow-up systems that extend beyond routine clinic visits through accessible counselling and continuity of care.

Financial issues were also a major issue reported by several participants. Many participants reported having to purchase warfarin using personal or family funds, often delaying or interrupting care due to the inability to afford travel, tests, or medication. The situation is worse for the majority of participants due to low income, since they lack health insurance, and therefore depend on out-of-pocket payments, illustrating the fragile continuum of care available to these patients, where treatment adherence becomes contingent on financial capacity rather than clinical need [36]. Findings from the study done at JKCI revealed the same issue, as some patients on warfarin missed appointments as a result of a lack of transport fare and a lack of money to cover warfarin costs [33]. Findings from a study done in Ethiopia [16] and other SSA countries also pointed out the same issue of affordability and anticoagulation control, with warfarin being a challenge [11,34]. These differences further underscore the need for strengthening financial risk protection as central to building a supportive system for long-term anticoagulation care.

Another important finding was the contradictory information participants received from healthcare providers regarding warfarin dosage, timing, and dietary practices. This confusion and inconsistency highlight the lack of standardized warfarin treatment protocols and inadequate training among healthcare providers in managing patients on warfarin therapy. Such variability places patients in a vulnerable position where they are required to interpret and reconcile conflicting instructions, increasing the risk of dosing errors, poor adherence, and adverse outcomes such as over-anticoagulation. This finding highlights not only communication gaps but also systemic weaknesses in continuity of care for patients requiring long-term warfarin monitoring. Similar findings were also observed in studies done in Iran and India, where inconsistent counselling contributed to patient confusion regarding warfarin management [32,37]. Contrary to countries like Canada, there is a better integrated health system, with the use of standardized warfarin protocols and the use of shared electronic medical records that help in ensuring consistency in communication among HCPs and eventually minimize patient confusion and promote safe warfarin use in the long run [15,18]. While the lack of integrated electronic medical record systems may contribute to these inconsistencies in Tanzania, the findings may also reflect broader challenges, such as limited standardized anticoagulation protocols, inconsistent provider training, and weak communication between tertiary cardiac centers and peripheral healthcare facilities where patients occasionally seek care. However, considering resource constraints in Tanzania, we might opt for less expensive entry points such as the use of low-cost paper-based communication booklets, WhatsApp groups for provider coordination, and SMS reminders to patients. Furthermore, improving coordination between referral and peripheral facilities is essential for a safer and more reliable continuum of care for patients on long-term warfarin management.

### Study strengths and limitations

The strength of this study lies in its focus on the patient’s lived experiences of long-term warfarin use in a low-resource setting in Tanzania, a country where most previous studies have primarily focused on clinical outcomes rather than patient perspectives and experiences. These findings provide important insights for improving anticoagulation care, patient health education, and decentralization of warfarin service delivery in similar low-resource settings. However, the study should be interpreted in light of some important limitations. First, there is potential recall and social desirability bias due to reliance on self-reported data. This was mitigated by assuring participants of confidentiality, encouraging honest responses, and using probing techniques during interviews to enhance the credibility and depth of the information collected. Second, this qualitative study was conducted at a single national cardiac institute, which may limit the transferability of the findings to other healthcare settings. Although the national cardiac institute receives patients from diverse geographical regions across Tanzania, patients attending a national referral center may differ from those managed at regional or district hospitals in terms of disease severity, frequency of follow-up, and access to specialized anticoagulation care. Therefore, while the national referral role of JKCI enabled the study to capture a broad range of patient experiences from different parts of the country, the findings should be interpreted with consideration of the specialized nature of the study setting and may be most transferable to a similar specialized cardiac care context. Third, the study has methodological limitations related to the use of a semi-structured interview guide. Although the guide helped ensure that clinically important aspects of long-term warfarin therapy were explored, it included sensitizing domains that are already recognized in the literature, such as forgetfulness, side effects, dietary modification, financial burden, inconsistent education, access to medication, and INR monitoring. This may have increased the possibility that interviews confirmed known barriers rather than identifying entirely new experiences. This risk was minimized by framing questions in an open-ended manner, using probes flexibly, and encouraging participants to raise additional issues beyond those included in the interview guide. During analysis, codes, sub-categories, and categories were retained only when grounded in participants’ narratives. Therefore, the findings should be interpreted as descriptive qualitative accounts generated through a combined inductive–deductive qualitative content analysis, rather than as findings from a purely inductive qualitative design. Fourth, although interviews were conducted in Kiswahili to ensure clarity and comfort among study participants, the subsequent translation of transcripts into English for analysis and reporting may have led to minor loss of contextual meaning or misinterpretation. Coding and initial analysis were conducted using the original Kiswahili transcripts before translation into English to preserve participants’ intended meanings. In addition, translated transcripts and selected quotations were reviewed by another bilingual member of the research team to enhance accuracy and consistency of interpretation, although formal back-translation was not performed. Member checking was conducted by returning summarized findings to participants for validation, which strengthened credibility and ensured that interpretations accurately reflected participants’ experiences.

### Conclusion and recommendations

This study explored day-to-day experiences and challenges encountered by patients with prosthetic heart valves on long-term use of warfarin at Jakaya Kikwete Cardiac Institute. Even though participants acknowledged warfarin as a vital life-saving anticoagulant, its long-term use was associated with substantial lifestyle adjustments. including dietary modification, emotional stress, discomfort from perceived side effects, and concerns about maintaining therapeutic INR levels consistently. Study participants also encountered challenges such as poor access to INR monitoring services, unavailability of warfarin in peripheral areas, financial burden, and contradictory information from HCPs. These findings demonstrate that long-term warfarin adherence is influenced not only by individual patient behaviors but also by structural and health system-related barriers within the Tanzanian context. Therefore, to promote safe and more equitable warfarin anticoagulation management, healthcare systems need to invest in accessible INR monitoring services in all regions, warfarin availability and affordability, organized follow-up care, and patient health education with emphasis and active engagement of patients at each clinic visit.

The study recommends the need for a comprehensive approach to improve warfarin management. To address contradictory counselling and fragmented communication between healthcare providers, simple and low-cost communication strategies such as standardized paper-based anticoagulation booklets, shared clinic notes, and WhatsApp-based coordination among providers may improve continuity of care. To address missed appointments and poor follow-up, mobile phone-based SMS reminders and follow-up calls may serve as feasible interventions in low-resource settings. Strengthening patient education using culturally appropriate materials in local languages and reinforcing counselling at each clinic visit may also improve understanding of dietary consistency, medication adherence, and INR monitoring.

At the health system level, expanding access to INR testing services and improving warfarin availability in regional and district hospitals could reduce financial and geographical barriers to care. Where feasible, point-of-care INR monitoring devices may further improve timely anticoagulation monitoring in peripheral settings. In addition, the incorporation of full coverage for warfarin-related services into public insurance schemes may help reduce out-of-pocket costs among patients requiring lifelong anticoagulation therapy. Finally, future research should explore healthcare providers’ perspectives on anticoagulation management and evaluate the feasibility and effectiveness of low-cost digital follow-up strategies such as SMS reminders and mobile health-supported anticoagulation care in Tanzania and similar low-resource settings.
