# Supplementary material for: Day-to-day experiences and challenges of long-term warfarin use among patients with prosthetic heart valves at a National Cardiac Institute in Tanzania: A qualitative study
Source: PLoS One. 2026 Aug 5;21(8):e0355506. doi: 10.1371/journal.pone.0355506 (PMC13440872; doi:10.1371/journal.pone.0355506)
Supplement: Supplementary file 2 [file pone.0355506.s002.doc]

Response to editor and reviewers’ comments

| **Journal requirements: Academic Editor comments** | | | |
| --- | --- | --- | --- |
| **Section** | **Editorial`s comment** | **Response** | **Page inserted** |
| 1. | Please ensure that your manuscript meets PLOS ONE's style requirements, including those for file naming | Thank you for this comment. We have carefully revised the manuscript to ensure compliance with PLOS ONE style requirements, including appropriate file naming, manuscript formatting, section organization, table placement, and overall journal formatting guidelines | N/A |
| 2. | Please provide additional details regarding participant consent. In the ethics statement in the Methods and online submission information, please ensure that you have specified (1) whether consent was informed and (2) what type you obtained (for instance, written or verbal, and if verbal, how it was documented and witnessed). If your study included minors, state whether you obtained consent from parents or guardians. If the need for consent was waived by the ethics committee, please include this information. | Response: Thank you for this important comment. We have revised the ethics statement in the Methods section and submission information to clearly specify that written informed consent was obtained from all participants prior to data collection. We also clarified that participants were fully informed about the study objectives, procedures, confidentiality, voluntary participation, and their right to withdraw at any time without consequences. | Page 8,9 |
| 3. | Please remove any funding-related text from the manuscript and let us know how you would like to update your Funding Statement. Currently, your Funding Statement reads as follows:  “This study was funded by the Muhimbili University of Health and Allied Sciences, with BTM as a recipient. However, MUHAS had no role in the study design, data collection, data analysis, decision to publish, or preparation of the manuscript.” Please include your amended statements within your cover letter; we will change the online submission form on your behalf. | Response: Thank you for this clarification. We have removed the funding-related statement from the manuscript text, including the Acknowledgments section, in accordance with PLOS ONE guidelines.  We wish the Funding Statement in the online submission system to read as follows:  “This study was funded by the Muhimbili University of Health and Allied Sciences (MUHAS) through institutional research support awarded to BTM. The co-authors affiliated with MUHAS contributed to the study in their academic and supervisory capacities and did not receive specific funding for this work. The funder had no role in the study design, data collection and analysis, decision to publish, or preparation of the manuscript. The specific roles of the authors are articulated in the ‘author contributions’ section.” | N/A |
| 4. | We note that the grant information you provided in the ‘Funding Information’ and ‘Financial Disclosure’ sections do not match.  When you resubmit, please ensure that you provide the correct grant numbers for the awards you received for your study in the ‘Funding Information’ section. | Thank you for bringing this to our attention. We would like to clarify that this study did not receive a specific research grant. Instead, the study received institutional research support from the Muhimbili University of Health and Allied Sciences (MUHAS) to support BTM’s Master’s research activities. We have carefully revised and aligned the information provided in both the ‘Funding Information’ and ‘Financial Disclosure’ sections to ensure consistency in the online submission system | N/A |
| 5. | Thank you for stating the following financial disclosure:  “This study was funded by the Muhimbili University of Health and Allied Sciences, with BTM as a recipient. However, MUHAS had no role in the study design, data collection, data analysis, decision to publish, or preparation of the manuscript.” We note that one or more of the authors is affiliated with the funding organization, indicating the funder may have had some role in the design, data collection, analysis or preparation of your manuscript for publication; in other words, the funder played an indirect role through the participation of the co-authors. If the funding organization did not play a role in the study design, data collection and analysis, decision to publish, or preparation of the manuscript and only provided financial support in the form of authors' salaries and/or research materials, please do the following: 1. Review your statements relating to the author contributions, and ensure you have specifically and accurately indicated the role(s) that these authors had in your study. These amendments should be made in the online form. 2. Confirm in your cover letter that you agree with the following statement, and we will change the online submission form on your behalf:  “The funder provided support in the form of salaries for authors [insert relevant initials], but did not have any additional role in the study design, data collection and analysis, decision to publish, or preparation of the manuscript. The specific roles of these authors are articulated in the ‘author contributions’ section. | Thank you for this clarification. We have carefully reviewed and updated the author contributions section in the online submission form to accurately reflect the specific roles of each author in the study.  We also confirm our agreement with the revised financial disclosure statement and wish the online submission system to reflect the following statement:  “This study was funded by the Muhimbili University of Health and Allied Sciences (MUHAS) through institutional research support awarded to BTM. The co-authors affiliated with MUHAS contributed to the study in their academic and supervisory capacities and did not receive specific funding for this work. The funder had no role in the study design, data collection and analysis, decision to publish, or preparation of the manuscript. The specific roles of the authors are articulated in the ‘author contributions’ section.” | N/A |
| 6. | We note that your Data Availability Statement is currently as follows: “All relevant data are within the manuscript and its Supporting Information files. Any additional data needed may be available upon request from the author”  Please confirm at this time whether or not your submission contains all raw data required to replicate the results of your study. Authors must share the “minimal data set” for their submission. PLOS defines the minimal data set to consist of the data required to replicate all study findings reported in the article, as well as related metadata and methods | Thank you for this comment. We confirm that the revised submission now includes the minimal data set required to replicate the findings of the study. The de-identified raw qualitative data supporting the findings, including relevant excerpts and supporting materials, will be uploaded as Supporting Information files in the revised submission.  We have also revised the Data Availability Statement to the following:  “All relevant data underlying the findings of this study are included within the manuscript and its Supporting Information files. De-identified raw qualitative data supporting the study findings have been provided as Supporting Information files as part of the revised submission.” | N/A |
| 7. | In the online submission form you indicate that your data is not available for proprietary reasons and have provided a contact point for accessing this data. Please note that your current contact point is a co-author on this manuscript. According to our Data Policy, the contact point must not be an author on the manuscript and must be an institutional contact, ideally not an individual. Please revise your data statement to a non-author institutional point of contact, such as a data access or ethics committee, and send this to us via return email. Please also include contact information for the third party organization, and please include the full citation of where the data can be found | Thank you for this important clarification. We have revised the Data Availability Statement and the online submission form accordingly.  We confirm that the data supporting the findings of this study will be fully available as Supporting Information files submitted with the revised manuscript. Therefore, the statement indicating restricted access due to proprietary reasons has been removed.  In line with PLOS ONE Data Policy, the previously indicated contact person (who is a co-author) has been replaced with an institutional point of contact. The designated data access contact is now the MUHAS Research Ethics Committee (institutional body), which may be reached for any data-related inquiries.  Contact details have been updated in the submission system to reflect this institutional committee as the appropriate data access point. All de-identified raw qualitative data and supporting materials will be included within the manuscript and its Supporting Information files, making the dataset directly accessible without requiring third-party request procedures. | N/A |
| 8. | If the reviewer comments include a recommendation to cite specific previously published works, please review and evaluate these publications to determine whether they are relevant and should be cited. There is no requirement to cite these works unless the editor has indicated otherwise. | Thank you for this guidance. We have carefully reviewed all reviewer-recommended references and evaluated their relevance to the manuscript. Only the publications that are directly relevant to the study context, objectives, and discussion have been incorporated into the revised manuscript. Irrelevant suggested citations have not been included, in accordance with the journal’s guidance that citations are not mandatory unless appropriate. | N/A |
| Reviewer #1 | | | |
| **Section** | **Reviewer`s comment** | **Response** | Page inserted |
| **Introduction** | The introduction is well-structured but contains some long sentences that could be broken down for better readability. For example, the last sentence of the introduction is very long and could be split to emphasize the research gap and the study's aim more clearly. | Thank you for this helpful suggestion. We have revised the final sentence of the introduction by breaking it into shorter, clearer sentences to improve readability and flow. In the revised version, we have separately emphasized the existing research gap and clearly stated the study aim, ensuring better clarity and alignment with the manuscript structure. | Page 4,5 |
| **Materials and Methods** | You mention "purposive sampling." Please provide a bit more detail on the specific criteria used. For example, did you purposefully select for variation in age, gender, time since surgery, or geographic location (e.g., rural vs. urban)? This would strengthen the credibility statement | We thank the reviewer for this important suggestion. We have now clarified the purposive sampling strategy in the Methods section by specifying that participants were selected to ensure variation in key characteristics, including age, gender, duration since valve surgery, type of prosthetic valve, and place of residence (urban vs. rural). This was done to capture a wide range of experiences with long-term warfarin use and strengthen the credibility and richness of the findings | Page 6 (line 126-129) |
|  | The description of the analysis is thorough, which is excellent. You could add a very brief statement about how you handled potential language nuances during the translation of codes and categories from Kiswahili to English, to further support confirmability. | We thank the reviewer for this valuable suggestion. We have now added a brief clarification in the Data Analysis section indicating that attention was paid to preserving meaning during translation from Kiswahili to English, with iterative checking of translated codes and categories against the original Kiswahili transcripts to ensure consistency and reduce loss of linguistic or contextual nuance, thereby strengthening confirmability. | Page 9 (line 193-202) |
| **Results** | The results are rich and compelling. The use of participant quotes is excellent. However, the introductory sentences to each sub-category are sometimes lengthy. Consider making them more direct. | We sincerely thank the Reviewer for this positive appraisal and helpful suggestion. We agree that some introductory sentences preceding sub-categories were unnecessarily lengthy. In response, we have revised these sections to make the introductory statements more concise and direct while maintaining clarity and coherence. We believe this improvement enhances readability and strengthens the presentation of the findings. | Page 11-18 |
|  | Address the "Contradictory Information" Theme More Deeply. This is a powerful and important finding | We sincerely thank the Reviewer for this important insight. We have strengthened the Discussion by expanding the interpretation of the “Contradictory Information” theme, highlighting how fragmented care and inconsistent communication across providers contribute to patient confusion, medication errors, and risks such as over-anticoagulation. We believe this revision enhances the analytical depth of the manuscript. | Page 17, 24,25 |
| **Discussions** | The discussion is currently very long, dense, and somewhat repetitive. It often reads more like a summary of the results with citations attached, rather than a deep, critical analysis of the findings in the context of existing literature. A more effective structure would be to frame the discussion around the two main categories that emerged from the results. | Thank you for this comment. The Discussion was substantially revised to improve clarity, conciseness, and analytical depth. Repetitive descriptions of findings were reduced, and the section was restructured around the two main categories that emerged from the results to provide a clearer and more interpretive narrative. Greater emphasis was placed on critical analysis of the findings within the Tanzanian and Sub-Saharan African context rather than restating results with citations. | Page 19-25 |
|  | In the discussion, you contrast it with countries using shared electronic records. While true, it's also worth considering if the issue might be deeper than just a lack of EMRs. Could it also stem from a lack of consensus on protocols among the specialists themselves, or a breakdown in communication between the tertiary center and peripheral clinics where patients might also seek care? This would add another layer of nuance to your analysis. | Thank you for this insightful comment. The Discussion was revised to provide a broader interpretation of inconsistent warfarin counselling beyond the absence of shared electronic medical records. Additional analysis was included to highlight potential systemic factors such as lack of standardized anticoagulation protocols, inconsistent provider training, and weak communication and coordination between tertiary cardiac centers and peripheral health facilities where patients may also seek care. This revision added greater contextual nuance to the interpretation of fragmented anticoagulation care pathways in Tanzania. |  |
| **Conclusion and recommendations** | The conclusion and recommendations are good but could be more tightly linked to the specific sub-categories in the findings. Create a clear, logical thread from the problem (finding) to the solution (recommendation). | Thank you for this constructive comment. We revised the Conclusion and Recommendations to align more closely with the identified sub-categories, creating a clearer link between the reported challenges and the proposed recommendations. | Page 26-27 |
| **Other(s)** | The manuscript would benefit significantly from a thorough edit for clarity, conciseness, and flow. | Done accordingly throughout the research document | N/A |
|  | A final, careful proofread is needed to catch minor grammatical errors and typos. |  |  |
| **Reviewer #2** | | | |
| **Section** | **Reviewer`s comment** | **Response** | **Page inserted** |
| **Title** | The title is overly long and contains redundant phrasing. The opening "Experiences with Warfarin Long-term Use" and the later "Day-to-day Experiences and Challenges" overlap in meaning. I suggest tightening it to something like "Day-to-day Experiences and Challenges of Long-term Warfarin Use among Patients with Prosthetic Heart Valves at a National Cardiac Institute in Tanzania: A Qualitative Study." | Thank you for this valuable suggestion. We agree that the original title contained overlapping phrasing and was unnecessarily lengthy. In response, we have revised the title to improve clarity, conciseness, and readability while maintaining the study's main focus.  The revised title now reads:  “Day-to-day Experiences and Challenges of Long-term Warfarin Use among Patients with Prosthetic Heart Valves at a National Cardiac Institute in Tanzania: A Qualitative Study.” | **Page 1** |
| **Abstract** | In the abstract, the Background (Lines 11–16) is disproportionately long relative to the Aim (Lines 17–19), and the transition between the two is abrupt. Consider closing the Background with one sentence explaining that quantitative studies cannot address the "how" and "why" of adherence, which would justify the qualitative aim. The Results section of the abstract (Lines 25–33) merely lists category and subcategory labels without conveying any substantive findings or illustrative quotations. Please add one to two sentences per category summarizing the core insight. The term "digital follow-ups" appears in the Conclusion (Line 37) but is scarcely developed in the main text (only Line 542 mentions it). Either expand this in the Discussion or remove it from the abstract. Finally, the Keywords (Line 39) include only three terms. Please add searchable terms such as "qualitative research," "medication adherence," "sub-Saharan Africa," and "Tanzania." | Thank you for these insightful comments. We have revised the abstract to improve clarity, balance, and consistency with the main manuscript. The Background section was shortened and refined, with an additional bridging sentence included to justify the qualitative approach by highlighting the limited ability of quantitative studies to explain the underlying “how” and “why” of warfarin adherence. The Results section was also revised to summarize the substantive findings under each category rather than only listing category labels. In addition, the statement regarding “digital follow-ups” was removed from the conclusion to maintain consistency with the scope of discussion in the manuscript. Lastly, the Keywords section was expanded by adding searchable terms including “qualitative research,” “medication adherence,” “sub-Saharan Africa,” and “Tanzania.” | **Page 1,2 (Line 10-45)** |
| **Methodological Label versus Actual Analytic Approach** | This is the most fundamental methodological concern in the manuscript and must be addressed carefully. The authors repeatedly describe the study as an "exploratory phenomenological design" in the Abstract (Line 20), and Study design (Lines 81–82). However, the Data Analysis section (Lines 146–147) applies Graneheim and Lundman's qualitative content analysis, which belongs to a different methodological tradition. Phenomenology, whether in the Husserlian descriptive tradition or Heideggerian interpretive tradition, seeks the essential structures of lived experience and uses analytic approaches such as Colaizzi, Giorgi, van Manen, or IPA, typically involving bracketing or epoché. Graneheim and Lundman's content analysis, by contrast, is a descriptive qualitative method with a different philosophical grounding and operational procedure. The authors must choose one position: if the actual method was content analysis, please revise Lines 20, and 81–82 to refer to the design as "an exploratory qualitative study using inductive content analysis" or "a qualitative descriptive design," and remove phenomenological language throughout; if phenomenology is to be retained, the analytic approach must be changed accordingly and the method section must describe bracketing and eidetic reduction. | Thank you for this important methodological observation. We agree that the previous description created inconsistency between the stated study design and the actual analytic approach used. In response, we revised the manuscript by removing the phenomenological terminology throughout the abstract and methods sections. The study is now consistently described as “an exploratory qualitative study using inductive content analysis,” which accurately reflects the methodological approach employed, including the use of Graneheim and Lundman’s qualitative content analysis framework. These revisions have been made accordingly throughout the manuscript. | **Page 1 (Line 21)**  **Page 5 (Line 98-102)**  **Page 9 (Line 191-223)** |
| **Introduction** | The introduction is generally well structured but contains several issues. Line 61 ends with two periods after "mandatory (10,12)..", which is an editorial error. Lines 66–72 cite quantitative adherence rates of 20–65%, yet the present study is qualitative and does not measure adherence. This creates an awkward juxtaposition between the quantitative framing of the problem and the qualitative aim of the study. I suggest rewriting Lines 73–78 to articulate the research gap more clearly: quantitative studies have documented low adherence as a phenomenon, but the question of why patients struggle to adhere and how adherence is negotiated in everyday life requires qualitative inquiry, which this study provides. The introduction also omits any reference to direct oral anticoagulants (DOACs). While mechanical valve patients continue to rely on warfarin as the standard of care, this clinical fact should be made explicit so readers understand why the study focuses on warfarin rather than newer alternatives. Furthermore, several key sub-Saharan Africa anticoagulation quality studies (for example, Mwita and colleagues) are cited in the Discussion but not introduced as background. Please move relevant citations forward. | Thank you for these important comments. We corrected the editorial error in the introduction and revised the final paragraphs to better clarify the research gap and align the quantitative background with the qualitative aim of the study. Specifically, we emphasized that previous quantitative studies have mainly documented the extent of poor adherence and associated factors, while limited evidence exists on how patients experience and negotiate long-term warfarin use in everyday life. We also added a statement clarifying that warfarin remains the standard anticoagulant therapy for patients with mechanical prosthetic heart valves despite the increasing use of DOACs in other cardiovascular conditions. In addition, relevant sub-Saharan Africa literature on anticoagulation control was incorporated into the background section to strengthen contextualization of the study. | Page 3,4,5 |
| **Materials and methods** | **Study design and sampling line**  Line 84 reads "The flexibility and subjectivity of this approach warranted a deep exploration," which frames subjectivity as a justification for the method. Subjectivity is a feature of qualitative research, not a rationale for it. Please revise to something like "The inductive and interpretive nature of this approach enabled an in-depth exploration...". The Sample size subsection (Lines 109–116) provides insufficient treatment of data saturation. The authors state that saturation was reached at ten participants and confirmed with two more, but do not specify the operational definition of saturation (code saturation versus meaning saturation), nor how "no new information" was systematically assessed (iteratively after each interview, or retrospectively after ten). Additionally, reference [21] cited at Line 110 is a quantitative study in Kenya and is not an appropriate methodological source for the principle of saturation. Please replace it with a methodological reference such as Saunders et al. (2018) or Hennink and Kaiser (2022). The inclusion and exclusion criteria (Lines 113–116) are embedded in a long sentence and should be separated into a dedicated "Inclusion and exclusion criteria" subsection for clarity. The specific purposive sampling strategy is also unclear: was maximum variation sampling used, and if so, along what dimensions (age, sex, duration of warfarin use, valve type, region of origin)? A sampling matrix would strengthen this section. Finally, the number of individuals approached who declined to participate (non-response rate) is not reported. This information is important for readers to judge sample representativeness. | We thank the reviewer for these important methodological comments. We have revised the methodology section accordingly. Specifically, the statement "The flexibility and subjectivity of this approach warranted a deep exploration," was refined to indicate that “the inductive and interpretive nature of this approach enabled an in-depth exploration” rather than framing subjectivity as the rationale for the method. The sample size subsection was expanded to clarify that both code and meaning saturation were achieved, and that saturation was assessed iteratively after each interview through continuous review of emerging codes, categories, and meanings. We also replaced the previous citation with a more appropriate methodological reference on data saturation. Furthermore, the inclusion and exclusion criteria were separated into a dedicated subsection for improved clarity. The purposive sampling strategy was further clarified as maximum variation purposive sampling based on age, sex, duration of warfarin use, type of prosthetic valve, and place of residence. In addition, we have now reported the number of participants approached and those who declined participation, including the reasons for non-participation, to improve transparency and allow readers to better judge sample representativeness. | Page 5 (line 100-102)  Page 6 (line 126-137)  Page 7(line 138-143) |
|  | **Data collection**  The timing of interviews (Lines 122–123, "the waiting time is usually two hours") raises a methodological concern. Conducting interviews while patients await their INR result may place them in a state of anxiety about the impending test, which could affect the openness and authenticity of their responses. Please discuss this in the Methods and address how it was mitigated. The manuscript also does not provide the interview guide or key probing topics. I recommend including the guide as a supplementary file, as this is a standard expectation under PLOS ONE policies and the COREQ checklist. The manuscript further lacks a statement of researcher reflexivity. What is the role and positionality of the first author (B.T.M.)? Is he a nurse employed at JKCI, a postgraduate student, or an external researcher? His relationship with participants (for example, whether he had previously provided care to any of them) could meaningfully shape interview dynamics. Please add a reflexivity paragraph describing researcher roles, prior assumptions, and how these were managed during data collection and analysis. | We have revised the Methods section to address all concerns raised. We clarified that although interviews were conducted during the waiting time for INR results due to clinic flow and patients’ travel constraints, measures were taken to minimize potential anxiety, including ensuring voluntary participation, informed consent, privacy, and the option to pause or stop the interview at any time. The interview guide has now been included as a supplementary file in accordance with PLOS ONE and COREQ requirements. We have also added a reflexivity statement describing the first author’s positionality as a postgraduate MSc Cardiovascular Nursing student during clinical rotation at the study site (OPD), with no prior caregiving relationship with participants, and explained how reflexivity was maintained throughout data collection and analysis. | Page 7(line 144-176) |
|  | **Ethical considerations**  Although ethical approval from MUHAS and hospital management is reported, the form of consent (written or oral) is not specified. This is information that PLOS ONE explicitly requires, so please add it around Lines 138–140. Given that several participants in Table 1 had only primary-level education (P01, P02, P04, P05, P07, P10), how was comprehension of the consent form ensured? Was a Kiswahili version of the consent form used? Was consent obtained orally with a witness present for those with limited literacy? Please clarify. | We have revised the Ethical Consideration section to explicitly specify that written informed consent was obtained from all participants, as required by PLOS ONE. We further clarified that for participants with limited literacy, consent information was provided and explained in Kiswahili to ensure full understanding prior to written consent, with additional clarification in simple language where needed. | Page 8 (line 177-189) |
|  | **Data analysis**  The description follows Graneheim and Lundman's standard steps and is broadly acceptable, but several clarifications are needed. The language in which coding was performed is not specified. Interviews were conducted in Kiswahili and the report is in English. Was coding done on Kiswahili transcripts, or after translation to English? This is a critical decision in cross-language qualitative research and has direct implications for the preservation of meaning. Please clarify around Lines 148–153. For methodological guidance on cross-language qualitative research, I recommend consulting Squires, A. (2009). Methodological challenges in cross-language qualitative research: A research review. International Journal of Nursing Studies, 46(2), 277–287 (DOI: 10.1016/j.ijnurstu.2008.08.006), which provides 14 widely used criteria for evaluating translation and interpretation in qualitative health research.  The iterative coding process between the PI and supervisors is described (Lines 157–174), but member checking is not mentioned. Was any mechanism used to return preliminary findings to participants for validation? If not, please acknowledge this as a limitation. Reference [26] (Graneheim and Lundman, 2004) is the foundational citation, but I strongly recommend adding the updated paper by the same research group: Graneheim UH, Lindgren BM, Lundman B. Methodological challenges in qualitative content analysis: A discussion paper. Nurse Educ Today. 2017 Sep;56:29-34. doi: 10.1016/j.nedt.2017.06.002. Epub 2017 Jun 17. PMID: 28651100. This 2017 paper provides clearer operational definitions of meaning unit, condensation, and abstraction, and distinguishes between manifest and latent content analysis, which would strengthen the methodological transparency of this section. | We thank the reviewer for these important methodological clarifications. We have now specified that coding and initial analysis were conducted in the original Kiswahili transcripts to preserve meaning, with selected excerpts translated into English for reporting. We also acknowledge and have added relevant methodological guidance on cross-language qualitative research (Squires, 2009) and strengthened the analytical rigor of the content analysis section by incorporating the updated methodological discussion by Graneheim, Lindgren & Lundman (2017). In addition, we have clarified that member checking was conducted by returning preliminary findings to selected participants to verify accuracy and ensure that interpretations reflected their intended meanings.  **Top of Form**  **Bottom of Form** | Page 9,10 (line 190-223) |
|  | **Trustworthiness**  Line 179 states that "The first author has a prolonged engagement in study settings," yet data collection lasted only two months (April to June 2025). Two months is difficult to characterize as prolonged engagement. Please clarify what is meant: did the first author work or train at JKCI prior to the study? If so, this prior relationship with the setting should be made explicit. Line 177 cites reference [27] (Kivunja and Kuyini) as the source for Lincoln and Guba's criteria, but that reference is an educational paradigms paper and is not appropriate here. Please cite the original monograph directly: Lincoln, Y. S., Guba, E. G. (1985). Naturalistic Inquiry. Sage Publications. The "audit trail" mentioned at Line 183 is not concretely demonstrated. I recommend providing an excerpt of the code book or a complete mapping of codes to subcategories to categories as a supplementary file to substantiate this claim. | We thank the reviewer for these important methodological clarifications. We have corrected the citation for Lincoln and Guba’s criteria by replacing the secondary source with the original reference (Lincoln & Guba, 1985). We have also clarified what was meant by “prolonged engagement” by specifying that the first author was a postgraduate MSc Cardiovascular Nursing student undertaking clinical rotation at the study site prior to and during data collection, which enabled contextual familiarity. In addition, we have strengthened the audit trail description by indicating that the codebook and an example of the coding framework have now been provided as supplementary material to enhance transparency and support confirmability. | Page 10,11 (line 224-243) |
| **Results** | **Participant Characteristics and Missing Clinical**  This is the most prominent clinical information gap in the manuscript. Table 1 reports only age, sex, education, occupation, and duration of warfarin use. It omits several clinical variables that are essential for interpreting the findings of a warfarin adherence study. Specifically, the following should be added: (a) valve type and position (mechanical versus bioprosthetic; mitral, aortic, or combined), as the authors themselves acknowledge at Lines 56–57 that target INR differs by valve position, making this variable indispensable; (b) underlying etiology (rheumatic, degenerative, or other); (c) most recent INR value or current therapeutic status; (d) daily warfarin dose range; (e) major comorbidities (atrial fibrillation, diabetes, hypertension); (f) history of bleeding or thromboembolic events; and (g) health insurance status, which is particularly important given that financial barriers are a major theme in the Discussion yet not visible in Table 1. Please revise the table and the accompanying descriptive text at Lines 193–197 to incorporate these variables. | We thank the reviewer for this important observation. We have revised Table 1 and the accompanying descriptive text to include additional clinical characteristics relevant to long-term warfarin use, including valve type and position, underlying etiology, INR therapeutic status, warfarin dosage, and health insurance status. These additions provide better clinical context for interpreting participants’ experiences and the challenges related to anticoagulation management**.** | Page 11,12 (line 245-255)  Page 35 |
|  | The two main category labels are overly long and conceptually underdeveloped. "A journey of acceptance, discomfort, relief, and modified lifestyle" (Line 202) packs four emotional descriptors into a single label, reading more like a concatenation of subcategories than an abstracted category. "Striving for warfarin therapeutic outcome within unstructured continuum of care" (Lines 202–203, 284–285) contains the valuable concept of "unstructured continuum of care," but "striving for therapeutic outcome" is clinically oriented in a way that does not fully align with a patient-centered qualitative study. Well-formulated category labels in qualitative research typically share several features: they are concise (generally no more than five to seven words), capture an abstracted insight rather than enumerate its components, use verb or noun phrases that convey analytic meaning, and resonate with the theoretical orientation of the study. I encourage the authors to revisit both category labels and reformulate them so that they are more concise and analytically evocative. | We sincerely thank the Reviewer for this constructive comment. We agree that the original category labels were overly descriptive and lacked sufficient conceptual conciseness. In response, we have revised the categories to improve clarity, abstraction, and analytical precision in line with qualitative research conventions. The category *“A journey of acceptance, discomfort, relief, and modified lifestyle”* has been revised to *“Adapting to life with long-term warfarin use,”* while *“Striving for warfarin therapeutic outcome within unstructured continuum of care”* has been revised to *“Navigating warfarin care within an unstructured continuum of care.”* We believe the revised labels are more concise, conceptually focused, and better capture the essence of participants’ experiences while maintaining a patient-centered qualitative orientation. | Page 12 (line 256,260)  Page 36 |
|  | In Table 2 (Lines 206–207), the abstraction level of the codes is inconsistent. Some entries are close to verbatim quotations (for example, "I can't even hold a machete," "I doubled warfarin dose myself"), while others sit at a descriptive or analytic level (for example, "I don't have health insurance," "I kept eating whole bowl of vegetables"). Drawing on Graneheim, Lindgren, and Lundman's (2017; see comment 7) discussion of abstraction levels, I recommend raising all codes to a consistent descriptive level. Verbatim quotations should be reserved for the narrative text or quotation boxes in the Results section rather than functioning as codes themselves. | We sincerely thank the Reviewer for this insightful methodological observation. We agree that the initial coding in Table 2 showed inconsistency in abstraction levels, where some codes were overly close to verbatim quotations while others were more descriptive or analytic. In response, we have revised Table 2 to ensure that all codes are consistently presented at a descriptive level, in line with Graneheim, Lindgren, and Lundman (2017) guidance on qualitative content analysis. Verbatim expressions have been removed from the coding column and retained only in the Results section as supporting quotations, thereby improving analytical clarity, consistency, and methodological rigor throughout the table. |  |
|  | Regarding internal inconsistencies in the quotations: P04 describes an INR of 11 discovered due to unexplained fatigue at Lines 260–262, and another INR of 11 attributed to twice-daily dosing at Lines 338–339. If these refer to the same event presented in two places, the repeated use may mislead readers into assuming that P04 had multiple severe episodes of over-anticoagulation. If they refer to two separate events, this suggests recurrent anticoagulation instability in this participant, which would have substantive implications for data interpretation. Please clarify the temporal relationship between these two narratives and, if appropriate, provide additional clinical context (this also reinforces the recommendation in comment 9 to include INR-related variables in Table 1). | We thank the reviewer for this important observation. We confirm that the two quotations referring to an INR of 11 in participant P04 described the same clinical episode. To avoid potential confusion or misinterpretation as separate events, we have removed the duplicate quotation and retained a single clear account of this episode in the Results section. This revision ensures consistency in presentation and improves clarity of the findings. | Page 17 (line 380-381) |
|  | A further point warrants deeper analytic engagement in the Discussion. P09's account contains a revealing tension: at Lines 329–331, this participant admits to continuing to eat vegetables that were restricted (reflecting patient agency), while at Lines 358–359 he describes regional unavailability of warfarin (reflecting structural constraint). The interplay between individual agency and structural barriers—in which patients are neither passive victims of the system nor fully unconstrained rational actors—is arguably one of the most analytically generative findings in this dataset. I recommend that the authors elevate this tension into a standalone analytic theme in the Discussion rather than leaving it as parallel observations in the Results. | We sincerely thank the Reviewer for this insightful comment. We agree that the tension between patient agency and structural constraints is an important analytic contribution. In response, we have incorporated this interpretation into the Discussion as a distinct analytic theme, highlighting how patients actively exercise agency in self-management while simultaneously navigating structural barriers such as limited access to warfarin. This revision strengthens the conceptual depth of the study by emphasizing the interaction between individual agency and health system constraints. | Page 23 (line 501-515) |
|  | Finally, please review the gender balance of quotations used throughout the Results. Quotations from P09 and P11 (both male participants) appear with relatively high frequency. I would encourage the authors to verify that the selected quotations adequately represent the range of perspectives across all twelve participants, with particular attention to whether the seven female participants, spanning different age groups and durations of warfarin use, are equally well represented in the narrative. | We sincerely thank the Reviewer for this important observation. We have revisited the Results section and rechecked all transcripts to ensure balanced representation of participants’ voices. In response, we have revised the narrative and replaced selected quotations to improve inclusion of female participants and ensure more even distribution of perspectives across all twelve participants. We believe these revisions enhance the credibility and representativeness of the findings. | Page 11-18 |
| **Discussions** | The Discussion suffers from excessive international comparison at the expense of local analytic depth. Nearly every subtheme includes comparisons to Kenya, Ethiopia, Botswana, Nigeria, Turkey, Iran, India, Canada, and the United Kingdom. The comparisons feel mechanical and dilute the analysis of the Tanzanian context. I recommend focusing the main comparisons on other sub-Saharan African health systems (Kenya, Ethiopia, Botswana), using Turkey and Iran as supplementary middle-income comparators, and referencing Canada or the United Kingdom only briefly as idealized benchmarks. Otherwise, the discussion implicitly suggests that Tanzania should replicate Canadian systems, which is neither feasible nor appropriate | Thank you for this valuable comment. More local analytical depth within Tanzania and comparable Sub-Saharan African settings was done. Comparisons with Kenya, Ethiopia, and Botswana were prioritized due to similarities in healthcare systems and anticoagulation challenges, while references to Turkey and Iran were retained only where directly relevant. Comparisons with high-income countries such as Canada and the United Kingdom were minimized to avoid unrealistic contextual implications. Greater emphasis was also placed on local structural barriers, including centralized anticoagulation services, limited INR monitoring access, financial constraints, and inconsistent counselling within the Tanzanian healthcare context. | Page 19-25 |
|  | On conceptual terminology: the phrase "holistic care" is used repeatedly (Lines 378, 389, 405, 420, 432) without definition. Please provide an operational definition or ground the term in an established framework such as the Chronic Care Model or person-centered care. More broadly, the Discussion lacks a theoretical framework. A stronger qualitative paper would employ a mid-range theoretical lens (for example, the Health Belief Model, Normalization Process Theory, Andersen's Behavioral Model of Health Services Use, or a candidacy framework) to organize the rich interview data into a more explanatory narrative. I encourage the authors to introduce at least one such lens. | Thank you for this comment. The Discussion was revised by removing repeated use of the term “holistic care” and replacing it with clearer terminology emphasizing supportive and patient-centered care, including integrated clinical monitoring, patient education, emotional support, and lifestyle counselling. In addition, Andersen’s Behavioral Model of Health Services Use was introduced as a guiding framework to strengthen the theoretical grounding of the Discussion by explaining how long-term warfarin adherence is shaped by the interaction between individual patient factors and broader health system constraints. | Page 19-21 |
| **Strengths and limitations** | Line 515 contains a typographical error: "ad transcripts" should read "and transcripts." The treatment of translation (Lines 512–517) is inadequate. The authors mention that one bilingual individual performed the translation, but do not address whether back-translation was performed, whether a second bilingual expert independently verified direct quotations, or in which language coding was carried out. These are central methodological issues in cross-language qualitative research and should be discussed in detail. Additionally, the single-site limitation (Lines 508–511) is acknowledged, but please also emphasize that JKCI is a national referral center, which means its patient population may differ from patients at district or regional hospitals in terms of disease severity and frequency of clinical contact. This has implications for the transferability of findings. The absence of member checking and limited researcher reflexivity should also be acknowledged here as methodological limitations. | Thank you for this insightful comment. We have revised the Strengths and Limitations section to provide a clearer and more rigorous description of the translation process, including analysis in Kiswahili, independent review of selected translations, and clarification that formal back-translation was not performed. We also added discussion of the single-site nature of JKCI as a national referral center and its implications for transferability, and explicitly included member checking as part of the credibility measures. Finally, we acknowledged the absence of detailed reflexivity reporting as a methodological limitation to strengthen transparency and rigor. | Page 25,26 (line 541-576) |
| **Conclusion** | The reference to "digital health tools" in the Conclusion (Line 542) is not supported by any substantive Discussion content. I recommend adding a paragraph that engages with existing evidence on SMS reminders and point-of-care INR devices in sub-Saharan Africa. | Thank you for this valuable comment. We addressed this by adding discussion content on pragmatic digital support strategies, including SMS reminders and point-of-care INR monitoring approaches reported in sub-Saharan Africa. We also revised the Conclusion to align with the Discussion by emphasizing feasible low-cost digital interventions that may strengthen follow-up, adherence, and continuity of anticoagulation care in resource-limited settings such as Tanzania. | Page 26 (line 577-590) |
| **Recommendations** | On the feasibility of recommendations: Lines 495–498 recommend standardized protocols, provider training, and interdisciplinary communication, and Lines 490–494 reference shared electronic medical records. These proposals may be unrealistic given Tanzania's current resource constraints. Please discuss pragmatic entry points more realistically, such as low-cost paper-based communication booklets, WhatsApp groups for provider coordination, or SMS-based reminders. | Thank you for this important comment. We revised the Recommendations to include more realistic and context-appropriate strategies for Tanzania, including paper-based communication booklets, WhatsApp coordination among healthcare providers, and SMS reminders for follow-up and adherence support, instead of relying mainly on resource-intensive electronic systems. These revisions strengthen the feasibility and applicability of the recommendations within low-resource settings. | Page 26,27 (line 578-608) |
| **References** | Reference formatting is inconsistent. Some entries include DOIs, others only URLs, and some use the format "Available from: 10.xxx" instead of proper DOI URLs. Please standardize to PLOS ONE's Vancouver style. Reference [27] (Kivunja and Kuyini) is an educational research paradigms paper and is not appropriate as a citation for Lincoln and Guba's trustworthiness criteria, as noted in comment 8. Please cite Lincoln and Guba (1985) directly. Reference [21] (Mariita et al.) is inappropriately used at Line 110 to support the principle of data saturation, as noted in comment 4, because it is a quantitative study. I also recommend adding Graneheim, Lindgren, and Lundman (2017) as an updated methodological reference, as specified in comment 7. | Thank you for this comment. We have standardized all references according to PLOS ONE Vancouver style, corrected DOI formatting, replaced inappropriate methodological citations (including Lincoln and Guba’s original source, 1985), corrected the citation used for data saturation with appropriate qualitative references, and added Graneheim, Lindgren, and Lundman (2017) to strengthen the methodological framework for content analysis. | Page 28-34 |
| **Language and Editorial Details** | In addition to the specific errors noted above, the manuscript would benefit from a careful language edit. Lines 5–6 contain a repeated word: "Muhimbili University of Health and Allied Sciences Sciences." Line 195 "More than half" would read more precisely as "Seven of the twelve participants (58%) were female." Line 198 table caption placement should follow PLOS ONE formatting requirements (typically above the table). | Thank you for this helpful comment. We have carefully proofread and corrected the manuscript to remove typographical errors, including the repeated word “Sciences” in the affiliation. We also improved clarity and precision of participant characteristics by revising the statement to “Seven of the twelve participants (58%) were female.” In addition, we corrected table formatting to align with PLOS ONE requirements by ensuring the table caption is placed above the table. | N/A |
| **Reporting Guidelines** | I strongly recommend that the authors use the COREQ (Consolidated criteria for Reporting Qualitative research) 32-item checklist to systematically review and strengthen reporting, and submit a completed COREQ checklist with the revised manuscript. The current manuscript is notably underreported in Domain 1 (Research team and reflexivity) and Domain 3 (Analysis and findings). COREQ provides a useful self-audit tool for addressing many of the issues raised in this review. | Thank you for this important recommendation. We have reviewed the manuscript using the COREQ (Consolidated Criteria for Reporting Qualitative Research) 32-item checklist to strengthen reporting quality, with particular attention to Domain 1 (research team and reflexivity) and Domain 3 (analysis and findings). We have revised the manuscript accordingly to improve transparency in methodological reporting and have prepared and will submit the completed COREQ checklist alongside the revised manuscript, as recommended. | N/A |
|  | | | |
