# Supplementary material for: Day-to-day experiences and challenges of long-term warfarin use among patients with prosthetic heart valves at a National Cardiac Institute in Tanzania: A qualitative study
Source: PLoS One. 2026 Aug 5;21(8):e0355506. doi: 10.1371/journal.pone.0355506 (PMC13440872; doi:10.1371/journal.pone.0355506)
Supplement: Supplementary file 3 [file pone.0355506.s003.doc]

Response to editor and reviewers’ comments

| **Journal requirements: Academic Editor comments** | | | |
| --- | --- | --- | --- |
| **Section** | **Editorial`s comment** | **Response** | **Page inserted** |
| 1. | Please review your reference list to ensure that it is complete and correct. If you have cited papers that have been retracted, please include the rationale for doing so in the manuscript text, or remove these references and replace them with relevant current references. Any changes to the reference list should be mentioned in the rebuttal letter that accompanies your revised manuscript | Thank you for this important comment. We have carefully reviewed and verified the full reference list to ensure that all references are complete, accurate, and correctly cited. We also checked the retraction status of all cited papers and confirmed that none of the references included in the manuscript have been retracted. Therefore, no references were removed, replaced, or updated in this revision, and no changes were made to the reference list. | N/A |
| **Reviewer #1** | | | |
| **Section** | **Reviewer`s comment** | **Response** | **Page inserted** |
| **General comment** | The authors have responded to the previous comments thoroughly. I have no further comments. Good luck | Thank you very much for your positive feedback and encouraging comments. We sincerely appreciate the reviewer’s careful assessment of our revised manuscript and are grateful that the previous comments have been adequately addressed | N/A |
| **Reviewer #2** | | | |
| **Section** | **Reviewer`s comment** | **Response** | **Page inserted** |
| **Results** | Table 1, two requested clinical variables still missing. Comorbidities (e.g., atrial fibrillation, diabetes, hypertension) and a history of bleeding or thromboembolic events were requested in the previous round but have not been added, and the response letter does not address their omission. The bleeding/thromboembolic history is particularly relevant given the prominence of over-anticoagulation and bleeding in the findings. Please add these variables or state explicitly that the data were not available. | Thank you for this important comment. We carefully re-assessed our interview data and confirmed that the requested clinical variables had been collected. We have now added comorbidities, particularly hypertension (HTN) and Diabetes Mellitus (DM), as well as a history of thromboembolic events, to Table 1 to improve the completeness of participants’ clinical characteristics. | Page 35 |
| **Discussion** | In the Discussion, the sentence “…regarding warfarin dosage, timing, and dietary practices. guidance. This confusion…” (Line 529) contains an orphaned word, “guidance.” Please delete it. | Thank you for pointing this out. We have deleted the orphaned word “guidance” from the sentence in the Discussion section to improve clarity and readability. | Page 24 (line 530) |
|  | The single-site limitation now contains two adjacent, contradictory statements: lines 561–565 argue that the referral-center population may limit transferability, while lines 565–567 argue that the diverse catchment improves transferability. Please integrate these into a single balanced statement. | Thank you for this helpful comment. We agree that the previous wording contained adjacent statements that could appear contradictory. We have revised and integrated the single-site limitation into one balanced statement, clarifying that although JKCI’s national referral role allowed us to capture diverse patient experiences from different geographical regions, the specialized nature of the setting may limit transferability to lower-level health facilities. | Page 25-26 (line 563-570) |
