# Supplementary material for: Day-to-day experiences and challenges of long-term warfarin use among patients with prosthetic heart valves at a National Cardiac Institute in Tanzania: A qualitative study
Source: PLoS One. 2026 Aug 5;21(8):e0355506. doi: 10.1371/journal.pone.0355506 (PMC13440872; doi:10.1371/journal.pone.0355506)
Supplement: Supplementary file 4 [file pone.0355506.s004.doc]

Response to editor comments

| **Academic Editor comments** | | | |
| --- | --- | --- | --- |
| **Section** | **Editorial's comment** | **Response** | **Page inserted** |
| 1. | The study does not fully demonstrate an inductive qualitative approach. The authors repeatedly state that they used inductive content analysis. However, the description of the interview guide and analytic process suggests that the study is better described as a directed or descriptive qualitative study rather than a purely inductive analysis. | Thank you for this important observation. We agree that the previous wording overstated the inductive nature of the study. In the revised manuscript, we have removed references suggesting that the study was purely inductive and have revised the study design and data analysis sections to describe the study as an exploratory descriptive qualitative study using qualitative content analysis with a combined inductive-deductive approach. We clarified that the deductive component was reflected in the use of broad sensitizing domains in the semi-structured interview guide, while the inductive component was reflected in the development of codes, sub-categories, and categories from participants’ narratives rather than from fixed predetermined coding categories | Page 2 (line 25-26)  Page 5 (line 104-110)  Page 9,10 (206-209) |
| 2 | The interview guide contains numerous predetermined domains, including medication use, adherence, side effects, dietary modification, quality of life, motivators, forgetfulness, inadequate education, conflicting advice, access to medication, and INR monitoring. Interviews also included predetermined probing questions around these issues. These domains closely mirror the final categories that emerged. | Thank you for this observation. We have revised the methods section to clarify the role of the interview guide. The semi-structured interview guide included broad sensitizing domains informed by previous literature, clinical practice, and known challenges in long-term warfarin management. These domains were used to guide the interviews and ensure that clinically relevant aspects of warfarin therapy were explored. However, they were not used as fixed coding categories. Participants were encouraged to describe their experiences freely and to raise additional issues that were important to them. The final codes, sub-categories, and categories were developed from participants’ narratives during qualitative content analysis. | Page 7 (line 154-163)  Page 9,10 (line 206-209) |
| 3. | Confirmation bias: Because the interview guide explicitly explores recognized barriers such as forgetfulness, side effects, dietary restrictions, financial burden, inconsistent education, and access to INR monitoring, there is a risk that the interviews confirmed existing assumptions rather than identifying novel patient experiences | Thank you for highlighting this important methodological issue. We have acknowledged this as a limitation in the revised manuscript. We added that the use of sensitizing domains in the semi-structured interview guide may have increased the possibility of confirming known barriers rather than identifying entirely new experiences. To minimize this risk, questions were framed in an open-ended manner, probes were used flexibly, participants were encouraged to introduce additional issues beyond the guide, and codes, sub-categories, and categories were retained only when grounded in participants’ own narratives. We have therefore clarified that the findings should be interpreted as descriptive qualitative accounts generated through a combined inductive–deductive qualitative content analysis, rather than as findings from a purely inductive qualitative design. | Page 27,28 (line 609-620) |
| 4. | The Andersen Behavioural Model is introduced only in the Discussion. If this framework genuinely informed interpretation after the inductive analysis was completed, this is acceptable. However, if the framework influenced coding or interpretation during analysis, then the analysis was no longer purely inductive. | Thank you for this clarification. We have revised the manuscript to state explicitly that Andersen’s Behavioural Model was applied only after completion of the qualitative content analysis. It was not used to design the interview guide, code the data, or develop the sub-categories and categories. Instead, it was used in the Discussion as an interpretive lens to deepen understanding of how participants’ experiences with long-term warfarin therapy were shaped by interactions between individual factors, social circumstances, and health-system conditions, including access to INR monitoring, medication availability, financial constraints, and continuity of counselling. | Page 11 (line 237-241)  Page 20 (line 448-461) |
| 5. | The two major categories are useful but remain largely descriptive. Examples include dietary modification, symptom relief, financial burden, inconsistent information, and access barriers. These are important findings but they largely describe experiences rather than explaining the process by which patients negotiate long-term anticoagulation. | Thank you for this constructive comment. We have revised the results opening and discussion to better show the process by which patients negotiated long-term anticoagulation. The revised manuscript now explains that the two categories represent a continuing process in which participants balanced the perceived life-saving benefits of warfarin with the practical, emotional, dietary, financial, and health-system demands of lifelong anticoagulation. We have clarified that participants negotiated long-term warfarin therapy by adapting their eating practices, responding to perceived side effects, adjusting medication-taking routines, seeking care despite financial and geographical barriers, and making self-management decisions in the context of inconsistent information and limited access to anticoagulation services. | Page 13 (line 284-295)  Page 20-26 (443-590) |
| 6 | The explanation of saturation is improved. The authors should acknowledge that saturation is a judgement rather than an objectively measurable endpoint. | Thank you for this helpful suggestion. We have revised the saturation description to acknowledge that saturation is an analytical judgement rather than an objectively measurable endpoint. The revised manuscript now states that saturation was assessed iteratively during data collection and analysis, and that recruitment was stopped when the research team judged that additional interviews were no longer generating new codes, sub-categories, categories, or substantially different meanings relevant to the study aim. We also retained the explanation that saturation was judged after ten interviews, followed by two additional interviews to assess the consistency of the emerging categories. | Page 6,7 (line 137-147) |
| 7 | The manuscript states: “member checking was conducted by returning preliminary findings to selected participants.” Please specify how many participants, how they were selected, what feedback was obtained, and whether any findings changed following member checking. | Thank you for this observation. We have expanded the member checking description in the revised manuscript. Member checking was conducted with four purposively selected participants, representing approximately one-third of the study sample and reflecting variation in sex, duration of warfarin use, place of residence, health insurance status, and anticoagulation status. Summarized findings were shared with them in Kiswahili to confirm whether the categories and sub-categories reflected their experiences and whether any interpretation was inaccurate or incomplete. Participants confirmed that the findings were consistent with their experiences. Minor wording clarifications were made, but no new category or sub-category emerged following member checking. | Page 11 (line 242-249) |
