# Supplementary material for: Day-to-day experiences and challenges of long-term warfarin use among patients with prosthetic heart valves at a National Cardiac Institute in Tanzania: A qualitative study
Source: PLoS One. 2026 Aug 5;21(8):e0355506. doi: 10.1371/journal.pone.0355506 (PMC13440872; doi:10.1371/journal.pone.0355506)
Supplement: Supplementary file 1 [file pone.0355506.s001.docx]

Thank you for the opportunity to review this manuscript. The study addresses day-to-day experiences and challenges of long-term warfarin use among prosthetic heart valve patients in a low-resource setting in Tanzania, a topic with considerable clinical and public health relevance. It fills an important literature gap by capturing patient perspectives in sub-Saharan Africa, where most existing research on anticoagulation is quantitative and clinically focused. The interview data are rich, and several participant narratives vividly illustrate how patients negotiate anticoagulation within a fragmented health system. However, the manuscript has several issues that warrant careful revision, particularly regarding methodological consistency, conceptual clarity, completeness of clinical information, abstraction level of the findings, and depth of discussion.

1. Title and Abstract (Lines 1–39)

The title is overly long and contains redundant phrasing. The opening "Experiences with Warfarin Long-term Use" and the later "Day-to-day Experiences and Challenges" overlap in meaning. I suggest tightening it to something like "Day-to-day Experiences and Challenges of Long-term Warfarin Use among Patients with Prosthetic Heart Valves at a National Cardiac Institute in Tanzania: A Qualitative Study." In the abstract, the Background (Lines 11–16) is disproportionately long relative to the Aim (Lines 17–19), and the transition between the two is abrupt. Consider closing the Background with one sentence explaining that quantitative studies cannot address the "how" and "why" of adherence, which would justify the qualitative aim. The Results section of the abstract (Lines 25–33) merely lists category and subcategory labels without conveying any substantive findings or illustrative quotations. Please add one to two sentences per category summarizing the core insight. The term "digital follow-ups" appears in the Conclusion (Line 37) but is scarcely developed in the main text (only Line 542 mentions it). Either expand this in the Discussion or remove it from the abstract. Finally, the Keywords (Line 39) include only three terms. Please add searchable terms such as "qualitative research," "medication adherence," "sub-Saharan Africa," and "Tanzania."

2. Methodological Label versus Actual Analytic Approach (Lines 20–24, 80–85, 145–147)

This is the most fundamental methodological concern in the manuscript and must be addressed carefully. The authors repeatedly describe the study as an "exploratory phenomenological design" in the Abstract (Line 20), and Study design (Lines 81–82). However, the Data Analysis section (Lines 146–147) applies Graneheim and Lundman's qualitative content analysis, which belongs to a different methodological tradition. Phenomenology, whether in the Husserlian descriptive tradition or Heideggerian interpretive tradition, seeks the essential structures of lived experience and uses analytic approaches such as Colaizzi, Giorgi, van Manen, or IPA, typically involving bracketing or epoché. Graneheim and Lundman's content analysis, by contrast, is a descriptive qualitative method with a different philosophical grounding and operational procedure. The authors must choose one position: if the actual method was content analysis, please revise Lines 20, and 81–82 to refer to the design as "an exploratory qualitative study using inductive content analysis" or "a qualitative descriptive design," and remove phenomenological language throughout; if phenomenology is to be retained, the analytic approach must be changed accordingly and the method section must describe bracketing and eidetic reduction.

3. Introduction (Lines 40–78)

The introduction is generally well structured but contains several issues. Line 61 ends with two periods after "mandatory (10,12)..", which is an editorial error. Lines 66–72 cite quantitative adherence rates of 20–65%, yet the present study is qualitative and does not measure adherence. This creates an awkward juxtaposition between the quantitative framing of the problem and the qualitative aim of the study. I suggest rewriting Lines 73–78 to articulate the research gap more clearly: quantitative studies have documented low adherence as a phenomenon, but the question of why patients struggle to adhere and how adherence is negotiated in everyday life requires qualitative inquiry, which this study provides. The introduction also omits any reference to direct oral anticoagulants (DOACs). While mechanical valve patients continue to rely on warfarin as the standard of care, this clinical fact should be made explicit so readers understand why the study focuses on warfarin rather than newer alternatives. Furthermore, several key sub-Saharan Africa anticoagulation quality studies (for example, Mwita and colleagues) are cited in the Discussion but not introduced as background. Please move relevant citations forward.

4. Study Design and Sampling (Lines 80–116)

Line 84 reads "The flexibility and subjectivity of this approach warranted a deep exploration," which frames subjectivity as a justification for the method. Subjectivity is a feature of qualitative research, not a rationale for it. Please revise to something like "The inductive and interpretive nature of this approach enabled an in-depth exploration...". The Sample size subsection (Lines 109–116) provides insufficient treatment of data saturation. The authors state that saturation was reached at ten participants and confirmed with two more, but do not specify the operational definition of saturation (code saturation versus meaning saturation), nor how "no new information" was systematically assessed (iteratively after each interview, or retrospectively after ten). Additionally, reference [21] cited at Line 110 is a quantitative study in Kenya and is not an appropriate methodological source for the principle of saturation. Please replace it with a methodological reference such as Saunders et al. (2018) or Hennink and Kaiser (2022). The inclusion and exclusion criteria (Lines 113–116) are embedded in a long sentence and should be separated into a dedicated "Inclusion and exclusion criteria" subsection for clarity. The specific purposive sampling strategy is also unclear: was maximum variation sampling used, and if so, along what dimensions (age, sex, duration of warfarin use, valve type, region of origin)? A sampling matrix would strengthen this section. Finally, the number of individuals approached who declined to participate (non-response rate) is not reported. This information is important for readers to judge sample representativeness.

5. Data Collection (Lines 117–132)

The timing of interviews (Lines 122–123, "the waiting time is usually two hours") raises a methodological concern. Conducting interviews while patients await their INR result may place them in a state of anxiety about the impending test, which could affect the openness and authenticity of their responses. Please discuss this in the Methods and address how it was mitigated. The manuscript also does not provide the interview guide or key probing topics. I recommend including the guide as a supplementary file, as this is a standard expectation under PLOS ONE policies and the COREQ checklist. The manuscript further lacks a statement of researcher reflexivity. What is the role and positionality of the first author (B.T.M.)? Is he a nurse employed at JKCI, a postgraduate student, or an external researcher? His relationship with participants (for example, whether he had previously provided care to any of them) could meaningfully shape interview dynamics. Please add a reflexivity paragraph describing researcher roles, prior assumptions, and how these were managed during data collection and analysis.

6. Ethical Considerations (Lines 135–144)

Although ethical approval from MUHAS and hospital management is reported, the form of consent (written or oral) is not specified. This is information that PLOS ONE explicitly requires, so please add it around Lines 138–140. Given that several participants in Table 1 had only primary-level education (P01, P02, P04, P05, P07, P10), how was comprehension of the consent form ensured? Was a Kiswahili version of the consent form used? Was consent obtained orally with a witness present for those with limited literacy? Please clarify.

7. Data Analysis (Lines 145–174)

The description follows Graneheim and Lundman's standard steps and is broadly acceptable, but several clarifications are needed. The language in which coding was performed is not specified. Interviews were conducted in Kiswahili and the report is in English. Was coding done on Kiswahili transcripts, or after translation to English? This is a critical decision in cross-language qualitative research and has direct implications for the preservation of meaning. Please clarify around Lines 148–153. For methodological guidance on cross-language qualitative research, I recommend consulting Squires, A. (2009). Methodological challenges in cross-language qualitative research: A research review. International Journal of Nursing Studies, 46(2), 277–287 (DOI: 10.1016/j.ijnurstu.2008.08.006), which provides 14 widely used criteria for evaluating translation and interpretation in qualitative health research.

The iterative coding process between the PI and supervisors is described (Lines 157–174), but member checking is not mentioned. Was any mechanism used to return preliminary findings to participants for validation? If not, please acknowledge this as a limitation. Reference [26] (Graneheim and Lundman, 2004) is the foundational citation, but I strongly recommend adding the updated paper by the same research group: Graneheim UH, Lindgren BM, Lundman B. Methodological challenges in qualitative content analysis: A discussion paper. Nurse Educ Today. 2017 Sep;56:29-34. doi: 10.1016/j.nedt.2017.06.002. Epub 2017 Jun 17. PMID: 28651100. This 2017 paper provides clearer operational definitions of meaning unit, condensation, and abstraction, and distinguishes between manifest and latent content analysis, which would strengthen the methodological transparency of this section.

8. Trustworthiness (Lines 175–191)

Line 179 states that "The first author has a prolonged engagement in study settings," yet data collection lasted only two months (April to June 2025). Two months is difficult to characterize as prolonged engagement. Please clarify what is meant: did the first author work or train at JKCI prior to the study? If so, this prior relationship with the setting should be made explicit. Line 177 cites reference [27] (Kivunja and Kuyini) as the source for Lincoln and Guba's criteria, but that reference is an educational paradigms paper and is not appropriate here. Please cite the original monograph directly: Lincoln, Y. S., Guba, E. G. (1985). Naturalistic Inquiry. Sage Publications. The "audit trail" mentioned at Line 183 is not concretely demonstrated. I recommend providing an excerpt of the code book or a complete mapping of codes to subcategories to categories as a supplementary file to substantiate this claim.

9. Participant Characteristics and Missing Clinical Information (Table 1, Line 198)

This is the most prominent clinical information gap in the manuscript. Table 1 reports only age, sex, education, occupation, and duration of warfarin use. It omits several clinical variables that are essential for interpreting the findings of a warfarin adherence study. Specifically, the following should be added: (a) valve type and position (mechanical versus bioprosthetic; mitral, aortic, or combined), as the authors themselves acknowledge at Lines 56–57 that target INR differs by valve position, making this variable indispensable; (b) underlying etiology (rheumatic, degenerative, or other); (c) most recent INR value or current therapeutic status; (d) daily warfarin dose range; (e) major comorbidities (atrial fibrillation, diabetes, hypertension); (f) history of bleeding or thromboembolic events; and (g) health insurance status, which is particularly important given that financial barriers are a major theme in the Discussion yet not visible in Table 1. Please revise the table and the accompanying descriptive text at Lines 193–197 to incorporate these variables.

10. Results Presentation (Lines 199–365)

The two main category labels are overly long and conceptually underdeveloped. "A journey of acceptance, discomfort, relief, and modified lifestyle" (Line 202) packs four emotional descriptors into a single label, reading more like a concatenation of subcategories than an abstracted category. "Striving for warfarin therapeutic outcome within unstructured continuum of care" (Lines 202–203, 284–285) contains the valuable concept of "unstructured continuum of care," but "striving for therapeutic outcome" is clinically oriented in a way that does not fully align with a patient-centered qualitative study. Well-formulated category labels in qualitative research typically share several features: they are concise (generally no more than five to seven words), capture an abstracted insight rather than enumerate its components, use verb or noun phrases that convey analytic meaning, and resonate with the theoretical orientation of the study. I encourage the authors to revisit both category labels and reformulate them so that they are more concise and analytically evocative.

In Table 2 (Lines 206–207), the abstraction level of the codes is inconsistent. Some entries are close to verbatim quotations (for example, "I can't even hold a machete," "I doubled warfarin dose myself"), while others sit at a descriptive or analytic level (for example, "I don't have health insurance," "I kept eating whole bowl of vegetables"). Drawing on Graneheim, Lindgren, and Lundman's (2017; see comment 7) discussion of abstraction levels, I recommend raising all codes to a consistent descriptive level. Verbatim quotations should be reserved for the narrative text or quotation boxes in the Results section rather than functioning as codes themselves.

Regarding internal inconsistencies in the quotations: P04 describes an INR of 11 discovered due to unexplained fatigue at Lines 260–262, and another INR of 11 attributed to twice-daily dosing at Lines 338–339. If these refer to the same event presented in two places, the repeated use may mislead readers into assuming that P04 had multiple severe episodes of over-anticoagulation. If they refer to two separate events, this suggests recurrent anticoagulation instability in this participant, which would have substantive implications for data interpretation. Please clarify the temporal relationship between these two narratives and, if appropriate, provide additional clinical context (this also reinforces the recommendation in comment 9 to include INR-related variables in Table 1).

A further point warrants deeper analytic engagement in the Discussion. P09's account contains a revealing tension: at Lines 329–331, this participant admits to continuing to eat vegetables that were restricted (reflecting patient agency), while at Lines 358–359 he describes regional unavailability of warfarin (reflecting structural constraint). The interplay between individual agency and structural barriers—in which patients are neither passive victims of the system nor fully unconstrained rational actors—is arguably one of the most analytically generative findings in this dataset. I recommend that the authors elevate this tension into a standalone analytic theme in the Discussion rather than leaving it as parallel observations in the Results.

Finally, please review the gender balance of quotations used throughout the Results. Quotations from P09 and P11 (both male participants) appear with relatively high frequency. I would encourage the authors to verify that the selected quotations adequately represent the range of perspectives across all twelve participants, with particular attention to whether the seven female participants, spanning different age groups and durations of warfarin use, are equally well represented in the narrative.

11. Discussion (Lines 366–498)

The Discussion suffers from excessive international comparison at the expense of local analytic depth. Nearly every subtheme includes comparisons to Kenya, Ethiopia, Botswana, Nigeria, Turkey, Iran, India, Canada, and the United Kingdom. The comparisons feel mechanical and dilute the analysis of the Tanzanian context. I recommend focusing the main comparisons on other sub-Saharan African health systems (Kenya, Ethiopia, Botswana), using Turkey and Iran as supplementary middle-income comparators, and referencing Canada or the United Kingdom only briefly as idealized benchmarks. Otherwise, the discussion implicitly suggests that Tanzania should replicate Canadian systems, which is neither feasible nor appropriate.

On conceptual terminology: the phrase "holistic care" is used repeatedly (Lines 378, 389, 405, 420, 432) without definition. Please provide an operational definition or ground the term in an established framework such as the Chronic Care Model or person-centered care. More broadly, the Discussion lacks a theoretical framework. A stronger qualitative paper would employ a mid-range theoretical lens (for example, the Health Belief Model, Normalization Process Theory, Andersen's Behavioral Model of Health Services Use, or a candidacy framework) to organize the rich interview data into a more explanatory narrative. I encourage the authors to introduce at least one such lens.

On the feasibility of recommendations: Lines 495–498 recommend standardized protocols, provider training, and interdisciplinary communication, and Lines 490–494 reference shared electronic medical records. These proposals may be unrealistic given Tanzania's current resource constraints. Please discuss pragmatic entry points more realistically, such as low-cost paper-based communication booklets, WhatsApp groups for provider coordination, or SMS-based reminders. The reference to "digital health tools" in the Conclusion (Line 542) is not supported by any substantive Discussion content. I recommend adding a paragraph that engages with existing evidence on SMS reminders and point-of-care INR devices in sub-Saharan Africa.

12. Strengths and Limitations (Lines 499–517)

Line 515 contains a typographical error: "ad transcripts" should read "and transcripts." The treatment of translation (Lines 512–517) is inadequate. The authors mention that one bilingual individual performed the translation, but do not address whether back-translation was performed, whether a second bilingual expert independently verified direct quotations, or in which language coding was carried out. These are central methodological issues in cross-language qualitative research and should be discussed in detail. Additionally, the single-site limitation (Lines 508–511) is acknowledged, but please also emphasize that JKCI is a national referral center, which means its patient population may differ from patients at district or regional hospitals in terms of disease severity and frequency of clinical contact. This has implications for the transferability of findings. The absence of member checking and limited researcher reflexivity should also be acknowledged here as methodological limitations.

13. Conclusion and Recommendations (Lines 518–543)

The Conclusion (Lines 518–529) and Recommendations (Lines 530–543) overlap substantially. I recommend either merging them or giving each a distinct function: the Conclusion should succinctly summarize findings in relation to the original research aim, while the Recommendations should focus on practice, policy, and future research. Line 542 recommends further research on digital health tools, echoing the abstract, but as noted above the main text does not substantively engage with this topic. Either develop the digital health discussion or remove the recommendation.

14. References (Lines 562–686)

Reference formatting is inconsistent. Some entries include DOIs, others only URLs, and some use the format "Available from: 10.xxx" instead of proper DOI URLs. Please standardize to PLOS ONE's Vancouver style. Reference [27] (Kivunja and Kuyini) is an educational research paradigms paper and is not appropriate as a citation for Lincoln and Guba's trustworthiness criteria, as noted in comment 8. Please cite Lincoln and Guba (1985) directly. Reference [21] (Mariita et al.) is inappropriately used at Line 110 to support the principle of data saturation, as noted in comment 4, because it is a quantitative study. I also recommend adding Graneheim, Lindgren, and Lundman (2017) as an updated methodological reference, as specified in comment 7.

15. Language and Editorial Details

In addition to the specific errors noted above, the manuscript would benefit from a careful language edit. Lines 5–6 contain a repeated word: "Muhimbili University of Health and Allied Sciences Sciences." Line 195 "More than half" would read more precisely as "Seven of the twelve participants (58%) were female." Line 198 table caption placement should follow PLOS ONE formatting requirements (typically above the table).

16. Reporting Guidelines

I strongly recommend that the authors use the COREQ (Consolidated criteria for Reporting Qualitative research) 32-item checklist to systematically review and strengthen reporting, and submit a completed COREQ checklist with the revised manuscript. The current manuscript is notably underreported in Domain 1 (Research team and reflexivity) and Domain 3 (Analysis and findings). COREQ provides a useful self-audit tool for addressing many of the issues raised in this review.

Conclusion：

This is a clinically and policy-relevant study that fills an important gap in the literature on patient experiences of long-term anticoagulation in sub-Saharan Africa. The findings on patient–system interactions, including self-adjustment of doses, dietary confusion, limited service access, and contradictory provider messaging, offer direct insights for practice. However, the inconsistency between methodological label and analytic approach, the absence of key clinical information, and the underreporting of reflexivity and translation procedures currently undermine the scholarly rigor of the paper. With careful attention to the revisions outlined above, this manuscript has strong potential to make a valuable contribution to anticoagulation care research in the region. I look forward to reading the revised version.
