# Supplementary material for: Day-to-day experiences and challenges of long-term warfarin use among patients with prosthetic heart valves at a National Cardiac Institute in Tanzania: A qualitative study
Source: PLoS One. 2026 Aug 5;21(8):e0355506. doi: 10.1371/journal.pone.0355506 (PMC13440872; doi:10.1371/journal.pone.0355506)
Supplement: Peer review history

# Peer review: Decision Letter 0

**DOI:** 10.1371/journal.pone.0355506.r001

**Authors:** Redoy Ranjan

21 Apr 2026

PONE-D-26-08352

Experiences with Warfarin Long-term Use: An Exploratory Phenomenological Study on Day-to-day Experiences and Challenges encountered by Patients with Prosthetic Heart Valves attending clinic at a National Cardiac Institute in Tanzania

PLOS One

Dear Dr. Mgala,

Thank you for submitting your manuscript to PLOS ONE. After careful consideration, we feel that it has merit but does not fully meet PLOS ONE’s publication criteria as it currently stands. Therefore, we invite you to submit a revised version of the manuscript that addresses the points raised during the review process.

==============================

**ACADEMIC EDITOR:**

The manuscript addresses a clinically relevant topic; however, significant concerns remain regarding methodological clarity, data analysis, and the robustness of the conclusions. Key issues include insufficient detail in the study design, limited adjustment for potential confounders, and the need for clearer presentation of results. Substantial revision is required to improve scientific rigour, transparency, and overall interpretability before the manuscript can be reconsidered.

==============================

Please submit your revised manuscript by Jun 05 2026 11:59PM. If you will need more time than this to complete your revisions, please reply to this message or contact the journal office at plosone@plos.org. When you're ready to submit your revision, log on to https://www.editorialmanager.com/pone/ and select the 'Submissions Needing Revision' folder to locate your manuscript file.

Please include the following items when submitting your revised manuscript:

- A letter that responds to each point raised by the academic editor and reviewer(s). You should upload this letter as a separate file labeled 'Response to Reviewers'.
- A marked-up copy of your manuscript that highlights changes made to the original version. You should upload this as a separate file labeled 'Revised Manuscript with Track Changes'.
- An unmarked version of your revised paper without tracked changes. You should upload this as a separate file labeled 'Manuscript'.

If you would like to make changes to your financial disclosure, please include your updated statement in your cover letter. Guidelines for resubmitting your figure files are available below the reviewer comments at the end of this letter.

If applicable, we recommend that you deposit your laboratory protocols in protocols.io to enhance the reproducibility of your results. Protocols.io assigns your protocol its own identifier (DOI) so that it can be cited independently in the future. For instructions see: https://journals.plos.org/plosone/s/submission-guidelines#loc-laboratory-protocols. Additionally, PLOS ONE offers an option for publishing peer-reviewed Lab Protocol articles, which describe protocols hosted on protocols.io. Read more information on sharing protocols at https://plos.org/protocols?utm_medium=editorial-email&utm_source=authorletters&utm_campaign=protocols.

As the corresponding author, your ORCID iD is verified in the submission system and will appear in the published article. PLOS supports the use of ORCID, and we encourage all coauthors to register for an ORCID iD and use it as well. Please encourage your coauthors to verify their ORCID iD within the submission system before final acceptance, as unverified ORCID iDs will not appear in the published article. *Only*  the individual author can complete the verification step; PLOS staff *cannot*  verify ORCID iDs on behalf of authors.

We look forward to receiving your revised manuscript.

Kind regards,

Mr Redoy Ranjan, MS (CV&TS), Ch.M. (Edin), PhD

Academic Editor

PLOS One

Journal Requirements:

When submitting your revision, we need you to address these additional requirements.

1. Please ensure that your manuscript meets PLOS ONE’s style requirements, including those for file naming. The PLOS ONE style templates can be found at

https://journals.plos.org/plosone/s/file?id=wjVg/PLOSOne_formatting_sample_main_body.pdf and

https://journals.plos.org/plosone/s/file?id=ba62/PLOSOne_formatting_sample_title_authors_affiliations.pdf

2. Please provide additional details regarding participant consent. In the ethics statement in the Methods and online submission information, please ensure that you have specified (1) whether consent was informed and (2) what type you obtained (for instance, written or verbal, and if verbal, how it was documented and witnessed). If your study included minors, state whether you obtained consent from parents or guardians. If the need for consent was waived by the ethics committee, please include this information.

If you are reporting a retrospective study of medical records or archived samples, please ensure that you have discussed whether all data were fully anonymized before you accessed them and/or whether the IRB or ethics committee waived the requirement for informed consent. If patients provided informed written consent to have data from their medical records used in research, please include this information.

3. Thank you for stating the following in the Acknowledgments Section of your manuscript:

“This study was funded by the Muhimbili University of Health and Allied Sciences, with BTM as a recipient. However, MUHAS had no role in the study design, data collection, data analysis, decision to publish, or preparation of the manuscript.”

We note that you have provided funding information that is not currently declared in your Funding Statement. However, funding information should not appear in the Acknowledgments section or other areas of your manuscript. We will only publish funding information present in the Funding Statement section of the online submission form.

Please remove any funding-related text from the manuscript and let us know how you would like to update your Funding Statement. Currently, your Funding Statement reads as follows:

“This study was funded by the Muhimbili University of Health and Allied Sciences, with BTM as a recipient. However, MUHAS had no role in the study design, data collection, data analysis, decision to publish, or preparation of the manuscript.”

Please include your amended statements within your cover letter; we will change the online submission form on your behalf.

4. We note that the grant information you provided in the ‘Funding Information’ and ‘Financial Disclosure’ sections do not match.

When you resubmit, please ensure that you provide the correct grant numbers for the awards you received for your study in the ‘Funding Information’ section.

5. Thank you for stating the following financial disclosure:

“This study was funded by the Muhimbili University of Health and Allied Sciences, with BTM as a recipient. However, MUHAS had no role in the study design, data collection, data analysis, decision to publish, or preparation of the manuscript.”

We note that one or more of the authors is affiliated with the funding organization, indicating the funder may have had some role in the design, data collection, analysis or preparation of your manuscript for publication; in other words, the funder played an indirect role through the participation of the co-authors. If the funding organization did not play a role in the study design, data collection and analysis, decision to publish, or preparation of the manuscript and only provided financial support in the form of authors' salaries and/or research materials, please do the following:

1. Review your statements relating to the author contributions, and ensure you have specifically and accurately indicated the role(s) that these authors had in your study. These amendments should be made in the online form.

2. Confirm in your cover letter that you agree with the following statement, and we will change the online submission form on your behalf:

“The funder provided support in the form of salaries for authors [insert relevant initials], but did not have any additional role in the study design, data collection and analysis, decision to publish, or preparation of the manuscript. The specific roles of these authors are articulated in the ‘author contributions’ section.

6. We note that your Data Availability Statement is currently as follows: “All relevant data are within the manuscript and its Supporting Information files. Any additional data needed may be available upon request from the author”

Please confirm at this time whether or not your submission contains all raw data required to replicate the results of your study. Authors must share the “minimal data set” for their submission. PLOS defines the minimal data set to consist of the data required to replicate all study findings reported in the article, as well as related metadata and methods (https://journals.plos.org/plosone/s/data-availability#loc-minimal-data-set-definition).

For example, authors should submit the following data:

- The values behind the means, standard deviations and other measures reported;

- The values used to build graphs;

- The points extracted from images for analysis.

Authors do not need to submit their entire data set if only a portion of the data was used in the reported study.

If your submission does not contain these data, please either upload them as Supporting Information files or deposit them to a stable, public repository and provide us with the relevant URLs, DOIs, or accession numbers. For a list of recommended repositories, please see https://journals.plos.org/plosone/s/recommended-repositories.

If there are ethical or legal restrictions on sharing a de-identified data set, please explain them in detail (e.g., data contain potentially sensitive information, data are owned by a third-party organization, etc.) and who has imposed them (e.g., an ethics committee). Please also provide contact information for a data access committee, ethics committee, or other institutional body to which data requests may be sent. If data are owned by a third party, please indicate how others may request data access.

7. In the online submission form you indicate that your data is not available for proprietary reasons and have provided a contact point for accessing this data. Please note that your current contact point is a co-author on this manuscript. According to our Data Policy, the contact point must not be an author on the manuscript and must be an institutional contact, ideally not an individual. Please revise your data statement to a non-author institutional point of contact, such as a data access or ethics committee, and send this to us via return email. Please also include contact information for the third party organization, and please include the full citation of where the data can be found.

8. If the reviewer comments include a recommendation to cite specific previously published works, please review and evaluate these publications to determine whether they are relevant and should be cited. There is no requirement to cite these works unless the editor has indicated otherwise.

[Note: HTML markup is below. Please do not edit.]

Reviewers' comments:

Reviewer’s Responses to Questions

**Comments to the Author**

1. Is the manuscript technically sound, and do the data support the conclusions?

The manuscript must describe a technically sound piece of scientific research with data that supports the conclusions. Experiments must have been conducted rigorously, with appropriate controls, replication, and sample sizes. The conclusions must be drawn appropriately based on the data presented.

Reviewer #1: Yes

Reviewer #2: Partly

**********

2. Has the statistical analysis been performed appropriately and rigorously?

Reviewer #1: Yes

Reviewer #2: N/A

**********

3. Have the authors made all data underlying the findings in their manuscript fully available?

The PLOS Data policy requires authors to make all data underlying the findings described in their manuscript fully available without restriction, with rare exception (please refer to the Data Availability Statement in the manuscript PDF file). The data should be provided as part of the manuscript or its supporting information, or deposited to a public repository. For example, in addition to summary statistics, the data points behind means, medians and variance measures should be available. If there are restrictions on publicly sharing data—e.g. participant privacy or use of data from a third party—those must be specified.

Reviewer #1: No

Reviewer #2: No

**********

4. Is the manuscript presented in an intelligible fashion and written in standard English?

PLOS ONE does not copyedit accepted manuscripts, so the language in submitted articles must be clear, correct, and unambiguous. Any typographical or grammatical errors should be corrected at revision, so please note any specific errors here.

Reviewer #1: No

Reviewer #2: Yes

**********

5. Review Comments to the Author

Please use the space provided to explain your answers to the questions above. You may also include additional comments for the author, including concerns about dual publication, research ethics, or publication ethics. (Please upload your review as an attachment if it exceeds 20,000 characters)

Reviewer #1: The manuscript addresses a relevant and important topic with a rigorous qualitative approach. The findings offer valuable insights into patient-level and health system-level barriers to warfarin adherence.

Major Issues

The discussion is currently very long, dense, and somewhat repetitive. It often reads more like a summary of the results with citations attached, rather than a deep, critical analysis of the findings in the context of existing literature. A more effective structure would be to frame the discussion around the two main categories that emerged from the results.

The manuscript would benefit significantly from a thorough edit for clarity, conciseness, and flow.

The introduction is well-structured but contains some long sentences that could be broken down for better readability. For example, the last sentence of the introduction is very long and could be split to emphasize the research gap and the study’s aim more clearly.

The results are rich and compelling. The use of participant quotes is excellent. However, the introductory sentences to each sub-category are sometimes lengthy. Consider making them more direct.

The conclusion and recommendations are good but could be more tightly linked to the specific sub-categories in the findings. Create a clear, logical thread from the problem (finding) to the solution (recommendation).

Minor issues

You mention "purposive sampling." Please provide a bit more detail on the specific criteria used. For example, did you purposefully select for variation in age, gender, time since surgery, or geographic location (e.g., rural vs. urban)? This would strengthen the credibility statement.

The description of the analysis is thorough, which is excellent. You could add a very brief statement about how you handled potential language nuances during the translation of codes and categories from Kiswahili to English, to further support confirmability.

Address the "Contradictory Information" Theme More Deeply. This is a powerful and important finding. In the discussion, you contrast it with countries using shared electronic records. While true, it’s also worth considering if the issue might be deeper than just a lack of EMRs. Could it also stem from a lack of consensus on protocols among the specialists themselves, or a breakdown in communication between the tertiary center and peripheral clinics where patients might also seek care? This would add another layer of nuance to your analysis.

A final, careful proofread is needed to catch minor grammatical errors and typos.

Reviewer #2: Dear Editor and Authors,

My full review exceeds the 20,000-character limit of this text box and has therefore been uploaded as an attachment. Please refer to the attached file for the complete review.

**********

6. PLOS authors have the option to publish the peer review history of their article (what does this mean?). If published, this will include your full peer review and any attached files.

If you choose “no”, your identity will remain anonymous but your review may still be made public.

**Do you want your identity to be public for this peer review?**  For information about this choice, including consent withdrawal, please see our Privacy Policy.

Reviewer #1: **Yes:**  Amr A. Arafat

Reviewer #2: No

**********

[NOTE: If reviewer comments were submitted as an attachment file, they will be attached to this email and accessible via the submission site. Please log into your account, locate the manuscript record, and check for the action link "View Attachments". If this link does not appear, there are no attachment files.]

To ensure your figures meet our technical requirements, please review our figure guidelines: https://journals.plos.org/plosone/s/figures

You may also use PLOS’s free figure tool, NAAS, to help you prepare publication quality figures: https://journals.plos.org/plosone/s/figures#loc-tools-for-figure-preparation.

NAAS will assess whether your figures meet our technical requirements by comparing each figure against our figure specifications.

Attachment Submitted filename: Comments.docx

# Author comment: Author response to Decision Letter 1

**DOI:** 10.1371/journal.pone.0355506.r002

29 May 2026

1. Academic Editor Comments

The Academic Editor raised nine administrative and ethical concerns related to PLOS ONE submission requirements. All have been addressed as described below.

1.1 Manuscript Style and Formatting

Comment: The manuscript did not fully comply with PLOS ONE style requirements including file naming and formatting.

Response: The manuscript was carefully revised to comply with all PLOS ONE style requirements, including file naming, manuscript formatting, section organization, and table placement.

1.2 Participant Consent

Comment: Additional detail was needed about consent type — whether informed, written or verbal — and how it was documented, especially for minors.

Response: The ethics statement was revised to specify that written informed consent was obtained from all participants. Details about voluntary participation, confidentiality, and right to withdraw were also added.

1.3 Funding Statement

Comment: Funding-related text needed to be removed from the manuscript and the Funding Statement updated in the submission system.

Response: Funding text was removed from the manuscript and the Acknowledgments section. An updated Funding Statement was submitted: MUHAS provided institutional research support to BTM; co-authors contributed in academic and supervisory capacities. The funder had no role in study design, data collection, analysis, or manuscript preparation.

1.4 Funding Information Discrepancy

Comment: Funding Information and Financial Disclosure sections were inconsistent.

Response: Both sections were revised and aligned to clarify that the study received institutional (not grant-based) support from MUHAS.

1.5 Author Affiliation with Funder

Comment: One or more authors were affiliated with the funding institution, suggesting indirect funder involvement.

Response: Author contributions were reviewed and updated in the submission system. The revised Financial Disclosure statement explicitly acknowledges that co-authors affiliated with MUHAS contributed in academic/supervisory capacities without direct funding.

1.6 Data Availability Statement

Comment: The data availability statement needed to confirm inclusion of the minimal dataset required to replicate study findings.

Response: The Data Availability Statement was revised to confirm that de-identified raw qualitative data supporting the findings have been provided as Supporting Information files.

1.7 Data Contact Person

Comment: The data contact point was a co-author, which is not permitted under PLOS ONE policy. An institutional (non-author) contact was required.

Response: The data contact was changed to the MUHAS Research Ethics Committee. All de-identified data are now included within the manuscript and Supporting Information files, removing the need for third-party requests.

1.8 Reviewer-Recommended Citations

Comment: Authors were reminded that reviewer-recommended citations are not mandatory.

Response: Only directly relevant recommended references were incorporated. Irrelevant suggestions were not included.

2. Reviewer #1 Comments

Reviewer #1 provided constructive feedback across all manuscript sections. Key revisions are summarized below.

2.1 Introduction - Sentence Length and Readability

Comment: Some sentences in the Introduction were too long and could be broken down for better readability, particularly the final sentence.

Response: The final sentences of the Introduction were restructured into shorter, clearer statements. The research gap and study aim are now stated separately for improved clarity.

2.2 Methods - Purposive Sampling Criteria

Comment: Insufficient detail was provided about the specific criteria used in purposive sampling (e.g., age, gender, location).

Response: The Methods section was revised to specify that maximum variation purposive sampling was used, capturing variation in age, gender, duration since valve surgery, prosthetic valve type, and urban vs. rural residence.

2.3 Methods - Language Translation and Confirmability

Comment: A statement was needed on how linguistic nuances were managed during translation from Kiswahili to English.

Response: A clarification was added to the Data Analysis section describing iterative checking of translated codes and categories against original Kiswahili transcripts to preserve meaning and support confirmability.

2.4 Results - Sub-Category Introductory Sentences

Comment: Introductory sentences to sub-categories were sometimes too lengthy.

Response: These sentences were revised to be more concise and direct while maintaining coherence.

2.5 Results - Contradictory Information Theme

Comment: The "Contradictory Information" theme warranted deeper analytic engagement in the Discussion.

Response: The Discussion was expanded to interpret how fragmented care and inconsistent provider communication contribute to patient confusion, medication errors, and over-anticoagulation risks.

2.6 Discussion - Structure and Analytical Depth

Comment: The Discussion was overly long, dense, and repetitive; it read more as a results summary with citations rather than a critical analysis.

Response: The Discussion was substantially restructured around the two main categories, with reduced repetition and greater emphasis on critical interpretation within the Tanzanian and sub-Saharan African context.

2.7 Discussion - Inconsistent Warfarin Counselling

Comment: Inconsistent counselling should be interpreted beyond the absence of electronic medical records, considering systemic factors.

Response: Analysis was expanded to include lack of standardized anticoagulation protocols, inconsistent provider training, and weak communication between tertiary and peripheral health facilities.

2.8 Conclusion and Recommendations

Comment: The Conclusion and Recommendations could be more tightly linked to specific sub-categories in the findings.

Response: Both sections were revised to create a clearer logical thread from identified challenges (findings) to proposed recommendations.

2.9 General Editing and Proofreading

Comment: The manuscript needed a thorough edit for clarity, conciseness, flow, and minor grammatical errors.

Response: Comprehensive language editing was carried out throughout the document.

3. Reviewer #2 Comments

Reviewer #2 provided extensive and detailed methodological, structural, and editorial feedback. The revisions made in response to this reviewer represent the most substantive changes in the manuscript.

3.1 Title

Comment: The title was overly long with overlapping phrasing.

Response: The title was revised to: "Day-to-day Experiences and Challenges of Long-term Warfarin Use among Patients with Prosthetic Heart Valves at a National Cardiac Institute in Tanzania: A Qualitative Study."

3.2 Abstract

Comment: The Background was disproportionately long; the Results section listed labels without substance; "digital follow-ups" was underdeveloped; and only three keywords were provided.

Response: The Abstract was revised to balance Background and Aim, strengthen the Results with substantive category summaries, remove "digital follow-ups" from the Conclusion, and expand Keywords to include qualitative research, medication adherence, sub-Saharan Africa, and Tanzania.

3.3 Methodological Label vs. Analytic Approach (Critical)

Comment: The manuscript inconsistently labelled the study as "exploratory phenomenological" while the analysis applied Graneheim and Lundman’s qualitative content analysis — a different methodological tradition.

Response: Phenomenological terminology was removed throughout. The study is now consistently described as "an exploratory qualitative study using inductive content analysis," accurately reflecting the approach used.

3.4 Introduction - Multiple Issues

Comment: Editorial errors, awkward juxtaposition of quantitative adherence rates and qualitative aim, absence of reference to DOACs, and key citations placed in the Discussion rather than Background.

Response: Errors were corrected; the research gap was reframed to emphasize that quantitative studies document low adherence while qualitative inquiry explains how and why; a statement on warfarin’s continued role in mechanical valve patients despite DOACs was added; and relevant sub-Saharan African anticoagulation literature was moved to the Background.

3.5 Methods - Study Design and Sampling

Comment: "Flexibility and subjectivity" misframed as a rationale; data saturation process was insufficiently described; inclusion/exclusion criteria were buried in a long sentence; purposive sampling strategy was unclear; non-response rate was not reported.

Response: Language was corrected to describe the "inductive and interpretive nature" of the approach. Saturation was clarified as both code and meaning saturation, assessed iteratively after each interview. Inclusion/exclusion criteria were separated into a dedicated subsection. Maximum variation sampling dimensions were specified. Non-response data were reported.

3.6 Methods - Data Collection and Timing

Comment: Conducting interviews while patients awaited INR results could affect openness due to anxiety; the interview guide was not provided; no reflexivity statement was included.

Response: Mitigation measures for interview timing were described. The interview guide was added as a supplementary file. A reflexivity paragraph was added describing the first author’s positionality as a postgraduate student during clinical rotation with no prior caregiving relationship with participants.

3.7 Methods - Ethical Considerations

Comment: The type of consent (written or verbal) was not specified; comprehension by participants with limited literacy was not addressed.

Response: Written informed consent was specified. For participants with limited literacy, the process of explaining the consent form in Kiswahili with additional clarification was described.

3.8 Methods - Data Analysis

Comment: The language of coding was not specified; member checking was absent; foundational citation needed updating; cross-language qualitative research guidance was needed.

Response: Coding was clarified as conducted on original Kiswahili transcripts with selected excerpts translated for reporting. Squires (2009) on cross-language methods and Graneheim, Lindgren & Lundman (2017) were added. Member checking with selected participants was described and reported.

3.9 Methods - Trustworthiness

Comment: "Prolonged engagement" was questionable, given the two-month data collection; Lincoln and Guba cited via an inappropriate secondary source; the audit trail was not demonstrated concretely.

Response: Prolonged engagement was clarified as arising from the first author’s clinical rotation at the site before and during data collection. Lincoln & Guba (1985) were cited directly. The codebook and coding framework were added as a supplementary file.

3.10 Results - Table 1: Missing Clinical Variables

Comment: Table 1 lacked essential clinical variables needed to interpret a warfarin adherence study (valve type/position, etiology, INR status, dosage, comorbidities, insurance status).

Response: Table 1 was revised to include valve type and position, underlying etiology, INR therapeutic status, daily warfarin dose range, and health insurance status.

3.11 Results - Category Labels

Comment: The two main category labels were overly long and analytically underdeveloped.

Response: Category labels were revised to: (1) "Adapting to life with long-term warfarin use" and (2) "Navigating warfarin care within an unstructured continuum of care."

3.12 Results - Table 2: Abstraction Level Inconsistency

Comment: Some codes were verbatim quotations rather than descriptive codes, creating inconsistency in abstraction level.

Response: Table 2 was revised so all codes are consistently presented at a descriptive level, per Graneheim, Lindgren & Lundman (2017). Verbatim expressions are retained only in the narrative Results section.

3.13 Results - Duplicate P04 Quotation

Comment: Participant P04’s INR of 11 appeared in two places, potentially implying two separate episodes.

Response: Confirmed as the same episode. The duplicate quotation was removed, retaining a single clear account.

3.14 Results - Agency vs. Structural Barriers

Comment: P09’s account revealed a tension between patient agency and structural constraints that warranted analytic elevation in the Discussion.

Response: This tension was incorporated into the Discussion as a distinct analytic theme, highlighting the interaction between individual agency and health system constraints in warfarin self-management.

3.15 Results - Gender Balance of Quotations

Comment: Male participants (P09, P11) were quoted with relatively high frequency; female participants needed equal representation.

Response: Quotations were revised to ensure more balanced representation across all twelve participants, with improved inclusion of female participants spanning different age groups and durations of warfarin use.

3.16 Discussion - Excessive International Comparisons

Comment: The Discussion over-relied on comparisons with high-income countries, diluting the local analytic depth.

Response: Main comparisons were refocused on sub-Saharan African health systems (Kenya, Ethiopia, Botswana). Middle-income comparators (Turkey, Iran) were retained only where directly relevant. High-income country comparisons were minimized.

3.17 Discussion - "Holistic Care" and Theoretical Framework

Comment: "Holistic care" was used repeatedly without definition; a theoretical framework was absent.

Response: "Holistic care" was replaced with precise patient-centered care terminology. Andersen’s Behavioral Model of Health Services Use was introduced as a guiding theoretical framework.

3.18 Strengths and Limitations - Translation, Single-Site, Member Checking

Comment: Translation process was inadequately described; single-site limitation needed elaboration; absence of member checking and limited reflexivity should be acknowledged.

Response: The translation process was described in detail, including Kiswahili-based coding and independent review of translations. JKCI’s national referral centre, nature, and implications for transferability were discussed. Member checking and limited reflexivity were acknowledged as limitations.

3.19 Conclusion - Digital Health Tools

Comment: Reference to "digital health tools" in the Conclusion was not substantiated by the Discussion content.

Response: A paragraph on evidence-based digital strategies (SMS reminders and point-of-care INR devices) in sub-Saharan Africa was added to the Discussion, and the Conclusion was aligned accordingly.

3.20 Recommendations - Feasibility

Comment: Recommendations for electronic medical records and standardized protocols were unrealistic given Tanzania’s resource constraints.

Response: Recommendations were revised to include pragmatic, context-appropriate strategies: paper-based communication booklets, WhatsApp provider coordination, and SMS reminders for follow-up and adherence.

3.21 References - Formatting and Appropriateness

Comment: Reference formatting was inconsistent; two inappropriate methodological citations were used; Graneheim et al. (2017) was absent.

Response: All references were standardized to PLOS ONE Vancouver style. Lincoln & Guba (1985) was cited directly. An appropriate saturation reference was substituted. Graneheim, Lindgren & Lundman (2017) was added.

3.22 Language and Editorial Details

Comment: Several minor errors: repeated "Sciences Sciences"; imprecise phrasing for gender count; table caption placement.

Response: All errors were corrected: repeated word removed; revised to "Seven of the twelve participants (58%) were female"; table captions placed above tables per PLOS ONE requirements.

3.23 COREQ Reporting Checklist

Comment: The COREQ 32-item checklist was recommended, particularly addressing Domain 1 (Research team and reflexivity) and Domain 3 (Analysis and findings).

Response: The manuscript was reviewed against the COREQ checklist. Deficiencies in Domains 1 and 3 were addressed, and the completed COREQ checklist is be submitted alongside the revised manuscript.

(A SEPARATE COMPREHENSIVE RESPONSE TO REVIEWER DOCUMENT HAS BEEN ATTACHED)

Attachment Submitted filename: Response to Reviewers.doc

# Peer review: Decision Letter 1

**DOI:** 10.1371/journal.pone.0355506.r003

**Authors:** Mergan Naidoo

18 Jun 2026

PONE-D-26-08352R1

Day-to-day Experiences and Challenges of Long-term Warfarin Use among Patients with Prosthetic Heart Valves at a National Cardiac Institute in Tanzania: A Qualitative Study

PLOS One

Dear Dr. Mgala,

Thank you for submitting your revised manuscript to PLOS ONE. After careful consideration, we feel that it has merit but one reviewer has suggested a minor revision. Therefore, we invite you to submit a revised version of the manuscript that addresses the points raised during the review process.

Please submit your revised manuscript by Aug 02 2026 11:59PM. If you will need more time than this to complete your revisions, please reply to this message or contact the journal office at plosone@plos.org. When you're ready to submit your revision, log on to https://www.editorialmanager.com/pone/ and select the 'Submissions Needing Revision' folder to locate your manuscript file.

Please include the following items when submitting your revised manuscript:

- A letter that responds to each point raised by the academic editor and reviewer(s). You should upload this letter as a separate file labeled 'Response to Reviewers'.
- A marked-up copy of your manuscript that highlights changes made to the original version. You should upload this as a separate file labeled 'Revised Manuscript with Track Changes'.
- An unmarked version of your revised paper without tracked changes. You should upload this as a separate file labeled 'Manuscript'.

If you would like to make changes to your financial disclosure, please include your updated statement in your cover letter. Guidelines for resubmitting your figure files are available below the reviewer comments at the end of this letter.

If applicable, we recommend that you deposit your laboratory protocols in protocols.io to enhance the reproducibility of your results. Protocols.io assigns your protocol its own identifier (DOI) so that it can be cited independently in the future. For instructions see: https://journals.plos.org/plosone/s/submission-guidelines#loc-laboratory-protocols. Additionally, PLOS ONE offers an option for publishing peer-reviewed Lab Protocol articles, which describe protocols hosted on protocols.io. Read more information on sharing protocols at https://plos.org/protocols?utm_medium=editorial-email&utm_source=authorletters&utm_campaign=protocols.

As the corresponding author, your ORCID iD is verified in the submission system and will appear in the published article. PLOS supports the use of ORCID, and we encourage all coauthors to register for an ORCID iD and use it as well. Please encourage your coauthors to verify their ORCID iD within the submission system before final acceptance, as unverified ORCID iDs will not appear in the published article. *Only*  the individual author can complete the verification step; PLOS staff *cannot*  verify ORCID iDs on behalf of authors.

We look forward to receiving your revised manuscript.

Kind regards,

Mergan Naidoo, PhD

Academic Editor

PLOS One

Journal Requirements:

If the reviewer comments include a recommendation to cite specific previously published works, please review and evaluate these publications to determine whether they are relevant and should be cited. There is no requirement to cite these works unless the editor has indicated otherwise.

Please review your reference list to ensure that it is complete and correct. If you have cited papers that have been retracted, please include the rationale for doing so in the manuscript text, or remove these references and replace them with relevant current references. Any changes to the reference list should be mentioned in the rebuttal letter that accompanies your revised manuscript. If you need to cite a retracted article, indicate the article’s retracted status in the References list and also include a citation and full reference for the retraction notice.

[Note: HTML markup is below. Please do not edit.]

Reviewers' comments:

Reviewer’s Responses to Questions

**Comments to the Author**

1. If the authors have adequately addressed your comments raised in a previous round of review and you feel that this manuscript is now acceptable for publication, you may indicate that here to bypass the “Comments to the Author” section, enter your conflict of interest statement in the “Confidential to Editor” section, and submit your "Accept" recommendation.

Reviewer #1: All comments have been addressed

Reviewer #2: (No Response)

**********

2. Is the manuscript technically sound, and do the data support the conclusions?

The manuscript must describe a technically sound piece of scientific research with data that supports the conclusions. Experiments must have been conducted rigorously, with appropriate controls, replication, and sample sizes. The conclusions must be drawn appropriately based on the data presented.

Reviewer #1: Yes

Reviewer #2: Yes

**********

3. Has the statistical analysis been performed appropriately and rigorously?

Reviewer #1: Yes

Reviewer #2: N/A

**********

4. Have the authors made all data underlying the findings in their manuscript fully available?

The PLOS Data policy requires authors to make all data underlying the findings described in their manuscript fully available without restriction, with rare exception (please refer to the Data Availability Statement in the manuscript PDF file). The data should be provided as part of the manuscript or its supporting information, or deposited to a public repository. For example, in addition to summary statistics, the data points behind means, medians and variance measures should be available. If there are restrictions on publicly sharing data—e.g. participant privacy or use of data from a third party—those must be specified.

Reviewer #1: No

Reviewer #2: Yes

**********

5. Is the manuscript presented in an intelligible fashion and written in standard English?

PLOS ONE does not copyedit accepted manuscripts, so the language in submitted articles must be clear, correct, and unambiguous. Any typographical or grammatical errors should be corrected at revision, so please note any specific errors here.

Reviewer #1: Yes

Reviewer #2: Yes

**********

6. Review Comments to the Author

Please use the space provided to explain your answers to the questions above. You may also include additional comments for the author, including concerns about dual publication, research ethics, or publication ethics. (Please upload your review as an attachment if it exceeds 20,000 characters)

Reviewer #1: The authors have responded to the previous comments thoroughly. I have no further comments.

Good luck

Reviewer #2: I thank the authors for a careful and substantive revision. The majority of the concerns raised in the previous round have been addressed in the manuscript text rather than only in the response letter, and the manuscript is clearly improved. A small number of issues remain. I therefore recommend a further minor revision, after which the manuscript should be suitable for publication.

1.Table 1, two requested clinical variables still missing. Comorbidities (e.g., atrial fibrillation, diabetes, hypertension) and a history of bleeding or thromboembolic events were requested in the previous round but have not been added, and the response letter does not address their omission. The bleeding/thromboembolic history is particularly relevant given the prominence of over-anticoagulation and bleeding in the findings. Please add these variables or state explicitly that the data were not available.

2. In the Discussion, the sentence “…regarding warfarin dosage, timing, and dietary practices. guidance. This confusion…” (Line 529) contains an orphaned word, “guidance.” Please delete it.

3.The single-site limitation now contains two adjacent, contradictory statements: lines 561–565 argue that the referral-center population may limit transferability, while lines 565–567 argue that the diverse catchment improves transferability. Please integrate these into a single balanced statement.

I thank the authors for their evident effort in revising this clinically and policy-relevant manuscript, and I look forward to seeing the minor points above resolved

**********

7. PLOS authors have the option to publish the peer review history of their article (what does this mean?). If published, this will include your full peer review and any attached files.

If you choose “no”, your identity will remain anonymous but your review may still be made public.

**Do you want your identity to be public for this peer review?**  For information about this choice, including consent withdrawal, please see our Privacy Policy.

Reviewer #1: **Yes:**  Amr A. Arafat

Reviewer #2: No

**********

[NOTE: If reviewer comments were submitted as an attachment file, they will be attached to this email and accessible via the submission site. Please log into your account, locate the manuscript record, and check for the action link "View Attachments". If this link does not appear, there are no attachment files.]

To ensure your figures meet our technical requirements, please review our figure guidelines: https://journals.plos.org/plosone/s/figures

You may also use PLOS’s free figure tool, NAAS, to help you prepare publication quality figures: https://journals.plos.org/plosone/s/figures#loc-tools-for-figure-preparation.

NAAS will assess whether your figures meet our technical requirements by comparing each figure against our figure specifications.

# Author comment: Author response to Decision Letter 2

**DOI:** 10.1371/journal.pone.0355506.r004

20 Jun 2026

We sincerely thank the Editor and Reviewers for their careful assessment of our revised manuscript and for the constructive comments that have helped us further improve the clarity and completeness of the paper. We have addressed all the minor comments as detailed below.

1. Editorial Comment: Please review your reference list to ensure that it is complete and correct. If you have cited papers that have been retracted, please include the rationale for doing so in the manuscript text, or remove these references and replace them with relevant current references. Any changes to the reference list should be mentioned in the rebuttal letter that accompanies your revised manuscript.

Response: Thank you for this important comment. We carefully reviewed and verified the full reference list to ensure that all references are complete, accurate, and correctly cited. We also checked the retraction status of all cited papers and confirmed that none of the references included in the manuscript have been retracted. Therefore, no references were removed, replaced, or updated in this revision, and no changes were made to the reference list.

Reviewer #1 Comment: The authors have responded to the previous comments thoroughly. I have no further comments. Good luck.

Response: Thank you very much for your positive feedback and encouraging comments. We sincerely appreciate the reviewer’s careful assessment of our revised manuscript and are grateful that the previous comments were considered adequately addressed.

Reviewer# 2 Comment 1: Table 1, two requested clinical variables still missing. Comorbidities, e.g., atrial fibrillation, diabetes, hypertension, and a history of bleeding or thromboembolic events were requested in the previous round but have not been added, and the response letter does not address their omission. The bleeding/thromboembolic history is particularly relevant given the prominence of over-anticoagulation and bleeding in the findings. Please add these variables or state explicitly that the data were not available.

Response: Thank you for this important comment. We carefully reassessed our interview data and confirmed that the requested clinical variables had been collected during the interviews. We have now added comorbidities and history of bleeding or thromboembolic events to Table 1 to improve the completeness of participants’ clinical characteristics. In the demographic and clinical characteristics section, we also added that five participants had previously experienced thromboembolic events, four participants had hypertension, and two participants had diabetes mellitus.

Reviewer #2 Comment 2: In the Discussion, the sentence “…regarding warfarin dosage, timing, and dietary practices. guidance. This confusion…” contains an orphaned word, “guidance.” Please delete it.

Response: Thank you for pointing this out. We have deleted the orphaned word “guidance” from the sentence in the Discussion section to improve clarity and readability.

Reviewer #2 Comment 3: The single-site limitation now contains two adjacent, contradictory statements: lines 561–565 argue that the referral-center population may limit transferability, while lines 565–567 argue that the diverse catchment improves transferability. Please integrate these into a single balanced statement.

Response: Thank you for this helpful comment. We agree that the previous wording contained adjacent statements that could appear contradictory. We have revised and integrated the single-site limitation into one balanced statement, clarifying that although JKCI’s national referral role allowed us to capture diverse patient experiences from different geographical regions, the specialized nature of the setting may limit transferability to lower-level health facilities.

Revised text in the manuscript: Second, this qualitative study was conducted at a single national cardiac institute, which may limit the transferability of the findings to other healthcare settings. Although the national cardiac institute receives patients from diverse geographical regions across Tanzania, patients attending a national referral center may differ from those managed at regional or district hospitals in terms of disease severity, frequency of follow-up, and access to specialized anticoagulation care. Therefore, while the national referral role of JKCI enabled the study to capture a broad range of patient experiences from different parts of the country, the findings should be interpreted with consideration of the specialized nature of the study setting and may be most transferable to a similar specialized cardiac care context.

Attachment Submitted filename: Response_to_Reviewers_auresp_2.doc

# Editor report: Decision Letter 2

**DOI:** 10.1371/journal.pone.0355506.r005

**Authors:** Mergan Naidoo

30 Jun 2026

PONE-D-26-08352R2

Day-to-day Experiences and Challenges of Long-term Warfarin Use among Patients with Prosthetic Heart Valves at a National Cardiac Institute in Tanzania: A Qualitative Study

PLOS One

Dear Dr. Mgala,

Thank you for submitting your manuscript to PLOS ONE. After careful consideration, we feel that it has merit but does not fully meet PLOS ONE’s publication criteria as it currently stands. Therefore, we invite you to submit a revised version of the manuscript that addresses the points raised during the review process.

Please submit your revised manuscript by Aug 14 2026 11:59PM. If you will need more time than this to complete your revisions, please reply to this message or contact the journal office at plosone@plos.org. When you're ready to submit your revision, log on to https://www.editorialmanager.com/pone/ and select the 'Submissions Needing Revision' folder to locate your manuscript file.

Please include the following items when submitting your revised manuscript:

- A letter that responds to each point raised by the academic editor and reviewer(s). You should upload this letter as a separate file labeled 'Response to Reviewers'.
- A marked-up copy of your manuscript that highlights changes made to the original version. You should upload this as a separate file labeled 'Revised Manuscript with Track Changes'.
- An unmarked version of your revised paper without tracked changes. You should upload this as a separate file labeled 'Manuscript'.

If you would like to make changes to your financial disclosure, please include your updated statement in your cover letter. Guidelines for resubmitting your figure files are available below the reviewer comments at the end of this letter.

We look forward to receiving your revised manuscript.

Kind regards,

Mergan Naidoo, PhD

Academic Editor

PLOS One

Journal Requirements:

If the reviewer comments include a recommendation to cite specific previously published works, please review and evaluate these publications to determine whether they are relevant and should be cited. There is no requirement to cite these works unless the editor has indicated otherwise.

Additional Editor Comments :

The study does not fully demonstrate an inductive qualitative approach: : The authors repeatedly state that they used ***inductive content analysis***  . However, the description of the interview guide and analytic process suggests that the study is better described as a directed or descriptive qualitative study rather than a purely inductive analysis. Specifically

- The interview guide contains numerous predetermined domains including medication use, adherence, side effects, dietary modification, quality of life, motivators, forgetfulness, inadequate education, conflicting advice, access to medication and INR monitoring.
- Interviews also included predetermined probing questions around these issues.
- These domains closely mirror the final categories that emerged

Confirmation bias: Because the interview guide explicitly explores recognised barriers (forgetfulness, side effects, dietary restrictions, financial burden, inconsistent education, access to INR monitoring), there is a risk that the interviews confirmed existing assumptions rather than identifying novel patient experiences.

The Andersen Behavioural Model is introduced only in the Discussion. If this framework genuinely informed interpretation after the inductive analysis was completed, this is acceptable. However, if the framework influenced coding or interpretation during analysis, then the analysis was no longer purely inductive.

The two major categories are useful but remain largely descriptive. Examples include:

- dietary modification
- symptom relief
- financial burden
- inconsistent information
- access barriers

These are important findings but they largely describe experiences rather than explaining the process by which patients negotiate long-term anticoagulation.

The explanation of saturation is improved. The authors should acknowledge that saturation is a judgement rather than an objectively measurable endpoint.

The manuscript states: "member checking was conducted by returning preliminary findings to selected participants."

Please specify:

- how many participants;
- how they were selected;
- what feedback was obtained;
- whether any findings changed following member checking

[Note: HTML markup is below. Please do not edit.]

[NOTE: If reviewer comments were submitted as an attachment file, they will be attached to this email and accessible via the submission site. Please log into your account, locate the manuscript record, and check for the action link "View Attachments". If this link does not appear, there are no attachment files.]

To ensure your figures meet our technical requirements, please review our figure guidelines: https://journals.plos.org/plosone/s/figures

You may also use PLOS’s free figure tool, NAAS, to help you prepare publication quality figures: https://journals.plos.org/plosone/s/figures#loc-tools-for-figure-preparation.

NAAS will assess whether your figures meet our technical requirements by comparing each figure against our figure specifications.

# Author comment: Author response to Decision Letter 3

**DOI:** 10.1371/journal.pone.0355506.r006

1 Jul 2026

Comment 1

Academic editor’s comment: The study does not fully demonstrate an inductive qualitative approach. The authors repeatedly state that they used inductive content analysis. However, the description of the interview guide and analytic process suggests that the study is better described as a directed or descriptive qualitative study rather than a purely inductive analysis.

Authors’ response: Thank you for this important observation. We agree that the previous wording overstated the inductive nature of the study. In the revised manuscript, we have removed references suggesting that the study was purely inductive and have revised the study design and data analysis sections to describe the study as an exploratory descriptive qualitative study using qualitative content analysis with a combined inductive-deductive approach. We clarified that the deductive component was reflected in the use of broad sensitizing domains in the semi-structured interview guide, while the inductive component was reflected in the development of codes, sub-categories, and categories from participants' narratives rather than from fixed predetermined coding categories. Page 2 (line 25-26), Page 5 (line 104-110), Page 9,10 (206-209)

Comment 2

Academic editor’s comment: The interview guide contains numerous predetermined domains including medication use, adherence, side effects, dietary modification, quality of life, motivators, forgetfulness, inadequate education, conflicting advice, access to medication, and INR monitoring. Interviews also included predetermined probing questions around these issues. These domains closely mirror the final categories that emerged.

Authors’ response: Thank you for this observation. We have revised the methods section to clarify the role of the interview guide. The semi-structured interview guide included broad sensitizing domains informed by previous literature, clinical practice, and known challenges in long-term warfarin management. These domains were used to guide the interviews and ensure that clinically relevant aspects of warfarin therapy were explored. However, they were not used as fixed coding categories. Participants were encouraged to describe their experiences freely and to raise additional issues that were important to them. The final codes, sub-categories, and categories were developed from participants' narratives during qualitative content analysis. Page 7 (line 154-163), Page 9,10 (line 206-209)

Comment 3

Academic editor’s comment: Confirmation bias: Because the interview guide explicitly explores recognized barriers such as forgetfulness, side effects, dietary restrictions, financial burden, inconsistent education, and access to INR monitoring, there is a risk that the interviews confirmed existing assumptions rather than identifying novel patient experiences.

Authors’ response: Thank you for highlighting this important methodological issue. We have acknowledged this as a limitation in the revised manuscript. We added that the use of sensitizing domains in the semi-structured interview guide may have increased the possibility of confirming known barriers rather than identifying entirely new experiences. To minimize this risk, questions were framed in an open-ended manner, probes were used flexibly, participants were encouraged to introduce additional issues beyond the guide, and codes, sub-categories, and categories were retained only when grounded in participants' own narratives. We have therefore clarified that the findings should be interpreted as descriptive qualitative accounts generated through a combined inductive-deductive qualitative content analysis, rather than as findings from a purely inductive qualitative design. Page 27,28 (line 609-620)

Comment 4

Academic editor’s comment: The Andersen Behavioural Model is introduced only in the Discussion. If this framework genuinely informed interpretation after the inductive analysis was completed, this is acceptable. However, if the framework influenced coding or interpretation during analysis, then the analysis was no longer purely inductive.

Authors’ response: Thank you for this clarification. We have revised the manuscript to state explicitly that Andersen’s Behavioural Model was applied only after completion of the qualitative content analysis. It was not used to design the interview guide, code the data, or develop the sub-categories and categories. Instead, it was used in the Discussion as an interpretive lens to deepen understanding of how participants' experiences with long-term warfarin therapy were shaped by interactions between individual factors, social circumstances, and health-system conditions, including access to INR monitoring, medication availability, financial constraints, and continuity of counselling. Page 11 (line 237-241), Page 20 (line 448-461)

Comment 5

Academic editor’s comment: The two major categories are useful but remain largely descriptive. Examples include dietary modification, symptom relief, financial burden, inconsistent information, and access barriers. These are important findings but they largely describe experiences rather than explaining the process by which patients negotiate long-term anticoagulation.

Authors’ response: Thank you for this constructive comment. We have revised the results opening and discussion to better show the process by which patients negotiated long-term anticoagulation. The revised manuscript now explains that the two categories represent a continuing process in which participants balanced the perceived life-saving benefits of warfarin with the practical, emotional, dietary, financial, and health-system demands of lifelong anticoagulation. We have clarified that participants negotiated long-term warfarin therapy by adapting their eating practices, responding to perceived side effects, adjusting medication-taking routines, seeking care despite financial and geographical barriers, and making self-management decisions in the context of inconsistent information and limited access to anticoagulation services Page 13 (line 284-295), Page 20-26 (443-590)

Comment 6

Academic editor’s comment: The explanation of saturation is improved. The authors should acknowledge that saturation is a judgement rather than an objectively measurable endpoint.

Authors’ response: Thank you for this helpful suggestion. We have revised the saturation description to acknowledge that saturation is an analytical judgement rather than an objectively measurable endpoint. The revised manuscript now states that saturation was assessed iteratively during data collection and analysis, and that recruitment was stopped when the research team judged that additional interviews were no longer generating new codes, sub-categories, categories, or substantially different meanings relevant to the study aim. We also retained the explanation that saturation was judged after ten interviews, followed by two additional interviews to assess consistency of the emerging categories. Page 6,7 (line 137-147)

Comment 7

Academic editor’s comment: The manuscript states: 'member checking was conducted by returning preliminary findings to selected participants.' Please specify how many participants, how they were selected, what feedback was obtained, and whether any findings changed following member checking.

Authors’ response: Thank you for this observation. We have expanded the member checking description in the revised manuscript. Member checking was conducted with four purposively selected participants, representing approximately one-third of the study sample and reflecting variation in sex, duration of warfarin use, place of residence, health insurance status, and anticoagulation status. Summarized findings were shared with them in Kiswahili to confirm whether the categories and sub-categories reflected their experiences and whether any interpretation was inaccurate or incomplete. Participants confirmed that the findings were consistent with their experiences. Minor wording clarifications were made, but no new category or sub-category emerged following member checking. Page 11 (line 242-249)

Attachment Submitted filename: Response_to_Reviewers_auresp_3.doc

# Editor report: Decision Letter 3

**DOI:** 10.1371/journal.pone.0355506.r007

**Authors:** Mergan Naidoo

22 Jul 2026

Day-to-day Experiences and Challenges of Long-term Warfarin Use among Patients with Prosthetic Heart Valves at a National Cardiac Institute in Tanzania: A Qualitative Study

PONE-D-26-08352R3

Dear Dr. Benedicto Mgala

We’re pleased to inform you that your manuscript has been judged scientifically suitable for publication and will be formally accepted for publication once it meets all outstanding technical requirements.

Within one week, you’ll receive an e-mail detailing the required amendments. When these have been addressed, you’ll receive a formal acceptance letter and your manuscript will be scheduled for publication.

An invoice will be generated when your article is formally accepted. Please note, if your institution has a publishing partnership with PLOS and your article meets the relevant criteria, all or part of your publication costs will be covered. Please make sure your user information is up-to-date by logging into Editorial Manager at Editorial Manager® and clicking the ‘Update My Information' link at the top of the page. For questions related to billing, please contact billing support.

If your institution or institutions have a press office, please notify them about your upcoming paper to help maximize its impact. If they’ll be preparing press materials, please inform our press team as soon as possible -- no later than 48 hours after receiving the formal acceptance. Your manuscript will remain under strict press embargo until 2 pm Eastern Time on the date of publication. For more information, please contact onepress@plos.org.

Kind regards,

Mergan Naidoo, PhD

Academic Editor

PLOS One

Additional Editor Comments (optional):

Reviewers' comments:

# Editor report: Acceptance letter

**DOI:** 10.1371/journal.pone.0355506.r008

**Authors:** Mergan Naidoo

PONE-D-26-08352R3

PLOS One

Dear Dr. Mgala,

I'm pleased to inform you that your manuscript has been deemed suitable for publication in PLOS One. Congratulations! Your manuscript is now being handed over to our production team.

At this stage, our production department will prepare your paper for publication. This includes ensuring the following:

* All references, tables, and figures are properly cited

* All relevant supporting information is included in the manuscript submission,

* There are no issues that prevent the paper from being properly typeset

You will receive further instructions from the production team, including instructions on how to review your proof when it is ready. Please keep in mind that we are working through a large volume of accepted articles, so please give us a few days to review your paper and let you know the next and final steps.

Lastly, if your institution or institutions have a press office, please let them know about your upcoming paper now to help maximize its impact. If they'll be preparing press materials, please inform our press team within the next 48 hours. Your manuscript will remain under strict press embargo until 2 pm Eastern Time on the date of publication. For more information, please contact onepress@plos.org.

You will receive an invoice from PLOS for your publication fee after your manuscript has reached the completed accept phase. If you receive an email requesting payment before acceptance or for any other service, this may be a phishing scheme. Learn how to identify phishing emails and protect your accounts at https://explore.plos.org/phishing.

If we can help with anything else, please email us at customercare@plos.org.

Thank you for submitting your work to PLOS ONE and supporting open access.

Kind regards,

PLOS ONE Editorial Office Staff

on behalf of

Professor Mergan Naidoo

Academic Editor

PLOS One
